# Engineered inhaled nanocatalytic therapy for ischemic cerebrovascular disease by inducing autophagy of abnormal mitochondria

**DOI:** 10.1038/s41536-023-00315-1

**Published:** 2023-08-11

**Authors:** Deping Wang, Bowen Li, Shuchao Wang, Yingjian Hao, Hua Wang, Wei Sun, Jimin Cao, Xin Zhou, Bin Zheng

**Affiliations:** 1 Academy of Medical Engineering and Translational Medicine, Tianjin Key Laboratory of Brain Science and Neural Engineering, Xincheng Hospital of Tianjin University, Tianjin University, Tianjin, 300072 China; 2 Key Laboratory of Cellular Physiology, Ministry of Education, and the Department of Physiology, Shanxi Medical University, Taiyuan, 030001 China; 3 School of Pharmaceutical Science and Technology, Tianjin University, 92 Weijin Road, Nankai District, Tianjin, 300072 China

**Keywords:** Stroke, Hypoxic-ischaemic encephalopathy

## Abstract

Mitochondrial dysfunction and subsequent accumulation of reactive oxygen species (ROS) are key contributors to the pathology of ischemic cerebrovascular disease. Therefore, elimination of ROS and damaged mitochondria is crucial for the effective treatment of this disease. For this purpose, we designed an inhalation nanotherapeutic agent, P/D@Mn/Co_3_O_4_, to treat ischemic cerebrovascular disease. Mn/Co_3_O_4_ effectively removed excess ROS from cells, reduced acute cellular oxidative stress, and protected neural cells from apoptosis. Furthermore, it depleted the H^+^ surrounding mitochondria and depolarized the mitochondrial membrane potential, inducing mitophagy and eliminating abnormal mitochondria, thereby avoiding the continuous overproduction of ROS by eliminating the source of ROS regeneration. On intranasal administration, Mn/Co_3_O_4_ encapsulated by platelet membranes and 2,3-(dioxy propyl)-trimethylammonium chloride can bypass the blood–brain barrier, enter the brain through the trigeminal and olfactory pathways, and target inflammatory regions to remove ROS and damaged mitochondria from the lesion area. In rat models of stroke and vascular dementia, P/D@Mn/Co_3_O_4_ effectively inhibited the symptoms of acute and chronic cerebral ischemia by scavenging ROS and damaged mitochondria in the affected area. Our findings indicate that the nanotherapeutic agent developed in this study can be used for the effective treatment of ischemic cerebrovascular disease.

## Introduction

Ischemic cerebrovascular disease (ICVD) poses a serious threat to human health. It is clinically classified into acute and chronic ischemia. Acute ischemia often leads to stroke that is characterized by hemiparesis and speech impairment. In contrast, chronic ischemia often leads to a progressive decline in the central nervous system function, eventually causing progressive cognitive impairment such as vascular dementia (VaD)^1–7^. Mitochondrial dysfunction and high levels of reactive oxygen species (ROS) are the leading causes of the onset and progression of ICVD^8,9^. Nanoenzymes with natural enzymatic activities, such as those with catalase (CAT)-like activity, have attracted attention owing to their ability to effectively scavenge ROS inside and outside the cells. Recently, Ce-doped zeolite-based nanoenzymes (Ce/Zeo-NMs) have been reported to reduce cerebral ischemia-reperfusion injury by adsorbing excess zinc ions and scavenging ROS^10^. However, currently available nanoenzymes only focus on effectively catalyzing the breakdown of ROS in the brain, while neglecting the root cause of ROS production. Metabolism and breakdown of nanoenzymes lead to the production of ROS, resulting in the recurrence of inflammation^11,12^. Therefore, the development of a method that can effectively remove ROS from the brain and inhibit ROS production is necessary to prevent the recurrence of ICVD.

Mitophagy, a form of selective autophagy, is a newly discovered process for the selective removal of excess or damaged mitochondria by cells. It plays a vital role in regulating the number of mitochondria in cells and maintaining mitochondrial function and cellular homeostasis^13–15^. Mitochondrial membrane potential (MMP) depolarization can induce mitophagy. Moreover, hydrogen ion (H^+^) pumped out of the mitochondrial respiratory chain complex on the inner mitochondrial membrane into the mitochondrial matrix play a key role in maintaining MMP^16–19^. Depletion of H^+^ depolarizes MMP and induces mitophagy by inducing ubiquitination degradation of mitochondria to eliminate the abnormal mitochondria relative to the increased production of ROS^20^. Therefore, the development of a nanoenzyme that can breakdown ROS in tissues and depolarize MMP by interfering with the supply of H^+^ in the mitochondrial matrix, eventually inducing abnormal mitophagy via ubiquitination degradation of mitochindria is necessary to prevent ROS production and facilitate the treatment of neuroinflammatory diseases.

**Fig. 1:**
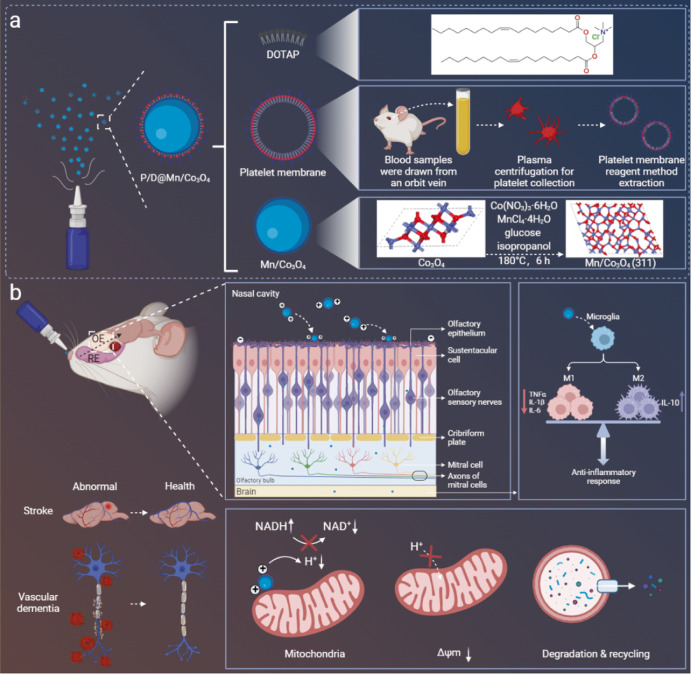
Synthesis, modification, and action mechanism of a nanotherapeutic agent in the amelioration of acute and chronic cerebral ischemic symptoms. **a** Synthesis of P/D@Mn/Co_3_O_4_. The synthesis process involves the preparation of Mn/Co_3_O_4_, extraction of platelet membranes, and preparation of P/D@Mn/Co_3_O_4_ by combining platelet membranes and 2,3-(dioxy propyl)-trimethylammonium chloride (DOTAP). **b** Mechanism of action of P/D@Mn/Co_3_O_4_ in vivo. The nanotherapeutic agent is delivered nasally and enters the brain via the trigeminal and olfactory pathways. Platelet membranes target the site of inflammation, while the positive charge imparted by DOTAP enhances the accumulation of P/D@Mn/Co_3_O_4_ around mitochondria owing to the strong electrostatic attraction. The enzyme-like activity of P/D@Mn/Co_3_O_4_ facilitates reactive oxygen species (ROS) scavenging, oxidative stress reduction, and prevention of neuronal cell apoptosis. By scavenging intracellular ROS, P/D@Mn/Co_3_O_4_ also depletes H^+^ around the mitochondria, reduces the supply of H^+^ to the mitochondrial matrix, and induces depolarization of the mitochondrial membrane potential (MMP), thereby causing mitotic and ubiquitinated degradation and preventing continuous ROS generation.

In this study, we designed a nanotherapeutic agent, P/D@Mn/Co_3_O_4_, from artificial platelet membranes and the cationic liposome, 2,3-(dioleoyloxy-propyl)-trimethylammonium-chloride (DOTAP), encapsulated in Mn/Co_3_O_4_. Nasal administration facilitates the easy entry of P/D@Mn/Co_3_O_4_ in the brain via the trigeminal and olfactory pathways, bypassing the blood–brain barrier (BBB). Under inflammatory conditions, endothelial cells become dysfunctional and platelets are mobilized to the site of inflammation. Artificial platelet membrane confers the ability to target the inflammatory regions of the brain to the nanotherapeutic agent. Moreover, DOTAP modification confers a strong positive charge to the surface of the nanotherapeutic agent, facilitating its strong electrostatic adsorption to the mitochondria of inflammatory cells after targeting the inflammatory region of the brain and reaching the outer mitochondrial membrane and nearby areas. After reaching the inflammatory regions of the brain, Mn/Co_3_O_4_ effectively scavenges ROS and induces autophagy of abnormal mitochondria. CAT-like activity of Mn/Co_3_O_4_ facilitaes ROS scavenging, oxidative stress alleviation, and prevention of neuronal cell apoptosis. Furthermore, Mn/Co_3_O_4_ scavenges ·OH and ·O^2−^ using its enzyme-like activity and consumes H^+^ around the mitochondria, thereby affecting the H^+^ supply within the mitochondrial matrix and MMP. Mn/Co_3_O_4_ eventually induces autophagy of abnormal mitochondria and eliminates ROS production, fundamentally solving the problem of excess ROS regeneration. Here, we constructed rat VaD and stroke models representing chronic and acute ICVD, respectively. We found that P/D@Mn/Co_3_O_4_ exerted neuroprotective effects and effectively alleviated VaD and stroke symptoms in vivo (Fig. 1). This study describes a previously unknown mechanism of ICVD and suggests a strategy for the treatment of other neuroinflammatory diseases and inflammatory disorders.

## Results

### Characterization and enzyme-mimicking activities of Mn/Co_3_O_4_ nanoparticles (NPs)

**Fig. 2:**
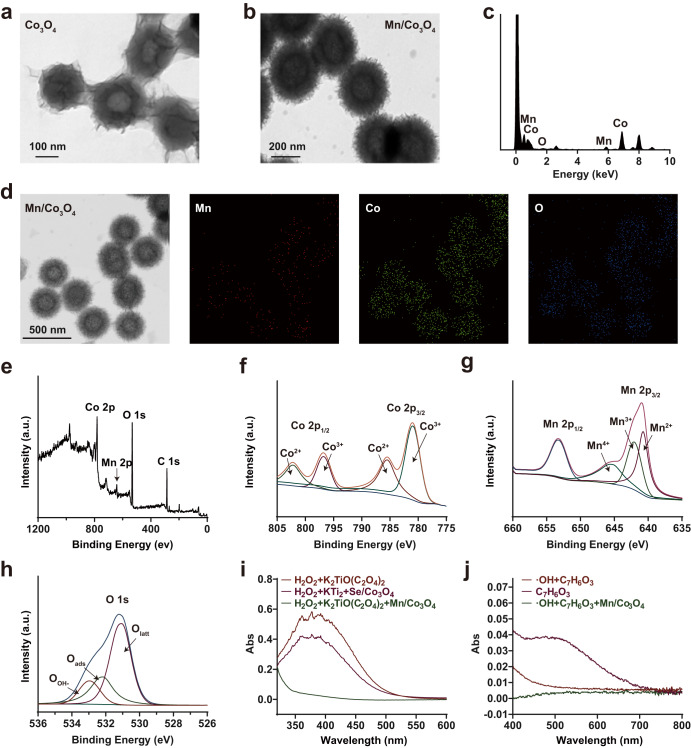
Characterization and enzyme-mimicking activities of Mn/Co_3_O_4_ nanoparticles (NPs). **a** Transmission electron microscopy (TEM) image of Co_3_O_4_ NPs (Scale bar: 100 nm). **b** TEM image of Mn/Co_3_O_4_ NPs (Scale bar: 200 nm). **c** TEM-energy dispersive spectroscopy (EDS) mapping of Mn/Co_3_O_4_. **d** TEM-EDS mapping of Mn/Co_3_O_4_ (Scale bar: 500 nm). **e** X-ray photoelectron spectroscopy (XPS) spectra of Mn/Co_3_O_4_. **f** XPS spectra of Co 2p for Mn/Co_3_O_4_. **g** XPS spectra of Mn 2p for Mn/Co_3_O_4_. **h** XPS spectra of O 1s for Mn/Co_3_O_4_. **i** Catalase (CAT)-like catalytic properties of Mn/Co_3_O_4_ NPs in the decomposition of hydrogen peroxide (H_2_O_2_). **j** Catalytic properties of Mn/Co_3_O_4_ NPs in the elimination of ·OH. *n* = 5 biologically independent samples in (**e**), (**f**), (**g**), (**h**), (**i**), and (**j**). Representative images of four biologically independent samples from each group are shown in (**a**), (**b**), (**c**), and (**d**).

In this study, a nanoenzyme was synthesized using a hydrothermal method. Transmission electron microscopy (TEM) images revealed that the particle size of Co_3_O_4_ NPs was approximately 200 nm, and the addition of Mn increased the particle size to approximately 300 nm and improved the dispersion of Mn/Co_3_O_4_ NPs (Fig. 2a, b). Dynamic light scattering measurements confirmed these observations (Supplementary Fig. 1). Energy spectrum analysis and mapping plots (Fig. 2c, d) revealed that the nanoenzyme contained three elements, Mn, Co, and O, with Mn being evenly dispersed throughout the nanocluster. The stability of the nanoenzyme was evaluated by measuring its UV absorption spectrum, which showed little change over two days, indicating its good stability (Supplementary Fig. 2). X-ray photoelectron spectroscopy (XPS) spectrum of Mn/Co_3_O_4_ NPs revealed that the nanoenzyme was primarily composed of Mn, Co, and O (Fig. 2e). The fine spectrum of Co (Fig. 2f) exhibited two main peaks centered at 779 and 795 eV, with a satellite peak at 804 eV. Peak-splitting analysis revealed two pairs of characteristic peaks of Co^3+^ and Co^2+^. Binding energies of 779 and 795 eV corresponded to the 2p_3/2_ and 2p_1/2_ orbitals of Co^3+^, respectively. The fine spectrum of Mn (Fig. 2g) exhibited two main peaks corresponding to the Mn 2p_3/2_ and Mn 2p_1/2_ orbitals, and peak splitting revealed the characteristic peaks of Mn^2+^, Mn^3+^, and Mn^4+^. The fine spectrum of O 1 s (Fig. 2h) exhibited three characteristic peaks obtained by splitting the peaks: surface hydroxyl oxygen $({\rm{O}}_{{\rm{OH}}^{-}})$ at 533.1 eV, surface adsorbed oxygen species (O_ads_) at 532.3 eV, and surface lattice oxygen (O_latt_) at 531.0 eV. XPS spectrum of Co_3_O_4_ NPs (Supplementary Fig. 3) revealed the presence of Co and O, but not Mn, similar to the spectrum og Mn/Co_3_O_4_ NPs. In conclusion, the nanoenzyme consisted of Co_3_O_4_ partially doped with Mn^2+^, Mn^3+^ and Mn^4+^. Different valence elements indicate the possibility of valence transformation, laying the foundation for its catalytic activity.

To investigate the enzyme-mimicking activities of our nanoenzyme in scavenging ROS, we performed relevant colorimetric experiments. As hydrogen peroxide (H_2_O_2_) and titanium ions form a stable orange complex in acidic media, potassium titanium oxalate was used as an indicator of H_2_O_2_^21^. Compared with the positive control group, the absorbance of the experimental group with Mn/Co_3_O_4_ NPs decreased significantly at 385 nm, indicating the elimination of ROS, such as H_2_O_2_, and confirming the CAT-like catalytic properties of Mn/Co_3_O_4_ NPs (Fig. 2i). This finding is consistent with previous reports demonstrating the CAT-like activity of Co_3_O_4_ in the decomposition of H_2_O_2_^22^. In particular, the addition of Mn further improved the catalytic activity of the nanoenzyme^23^. As salicylic acid can react with hydroxyl radicals (·OH) to produce dihydroxybenzoic acid, we used salicylic acid to indicate ·OH. We found that the absorbance of the experimental group with Mn/Co_3_O_4_ NPs at 510 nm was lower than that of the control group, which confirmed the elimination of ·OH (Fig. 2j). In addition, we investigated the changes in its catalytic ability based on the concentration and action time of Mn/Co_3_O_4_ NPs. The results revealed that its catalytic ability was enhanced with increasing concentration and action time of Mn/Co_3_O_4_ NPs (Supplementary Fig. 4 and 5). Notably, a moderate inhibitory effect was also observed, which may be affected by the amount of substrate.

### P/D@Mn/Co_3_O_4_ NPs decrease ROS levels, apoptosis, and inflammation in neuronal cells

**Fig. 3:**
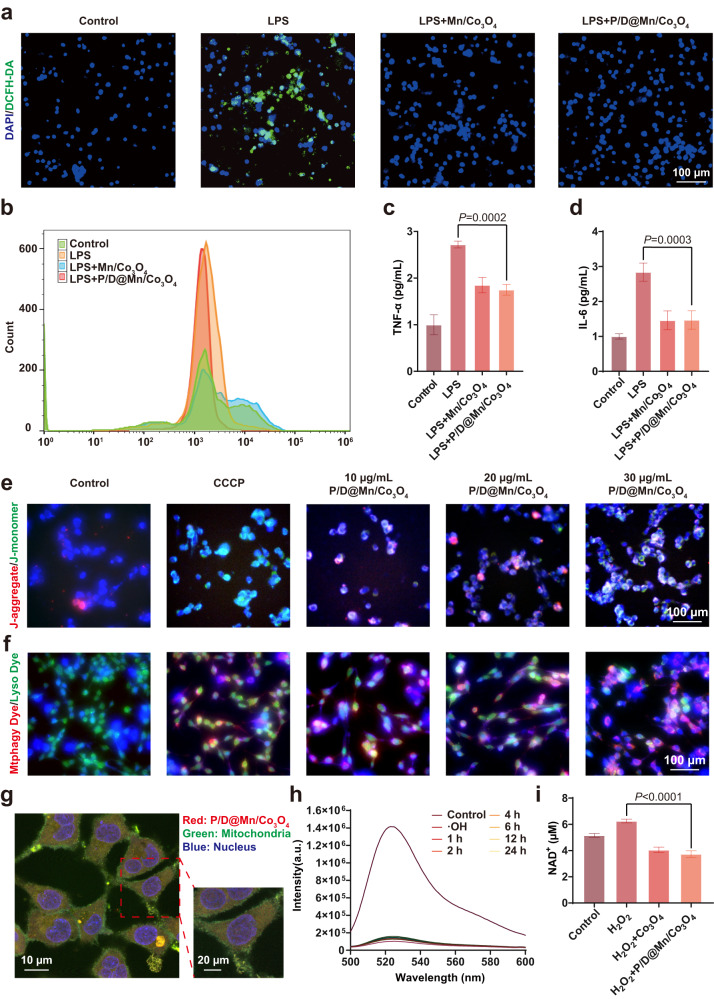
P/D@Mn/Co_3_O_4_ decreases ROS levels, apoptosis, and inflammation in neuronal cells and induces MMP depolarization and mitophagy. **a** Representative image of ROS levels in neuronal HT22 cells with different treatments via 2',7’-dichlorodihydrofluorescein diacetate (DCFH-DA) staining (Scale bar: 100 µm). **b** Flow cytometric analysis of ROS scavenging by Mn/Co_3_O_4_ and P/D@Mn/Co_3_O_4_. **c** Enzyme-linked immunosorbent assay (ELISA) to determine the secretion levels of tumor necrosis factor (TNF)-α in HT22 cells with different treatments. **d** ELISA to determine the secretion levels of interleukin (IL)-6 in HT22 cells with different treatments. **e** Confocal laser scanning microscopy (CLSM) for the determination of changes in MMP (Scale bar: 100 µm). **f** CLSM for the detection of mitophagy (Scale bar: 100 µm). **g** Co-localization image of P/D@Mn/Co_3_O_4_ and mitochondria (Scale bar: 10 µm; enlarged map scale bar: 20 µm). **h** ROS levels in cell supernatant after treatment with P/D@Mn/Co_3_O_4_ at different time points. **i** Levels of nicotinamide adenine dinucleotide (NAD^+^) in cells treated with the nanoenzyme after oxidative stress. Data in (**c**), (**d**), and (**i**) are represented as the mean values ± standard deviation (SD). One-way ANOVA with Bonferroni post hoc test for pair-wise comparisons. *P* > 0.05 "not significant"; *P* < = 0.05 "significant", *P* < = 0.01 means "very significant. *n* = 5 biologically independent samples in (**c**), (**d**), (**h**), and (**i**). Representative images of four biologically independent samples from each group are shown in (**a**), (**b**), (**e**), (**f**), and (**g**).

To demonstrate the effects of the nanoenzyme on organisms, we performed various in vitro cell experiments, which laid the foundation for subsequent animal experiments. First, we investigated the effects of different concentrations of Mn/Co_3_O_4_ and P/D@Mn/Co_3_O_4_ NPs on cell viability using the cell counting kit-8 (CCK-8) assay. We found that the cell mortality rate of both Mn/Co_3_O_4_ and P/D@Mn/Co_3_O_4_ NPs was less than 20% until the concentration reached 100 µg/mL, demonstrating the excellent biosafety of the nanoenzyme (Supplementary Fig. 6). We also performed Calcein acetoxymethyl ester (Calcein-AM)/Propidium iodide (PI) staining of cells to confirm their state (live or dead) after 24 h incubation with the nanoenzyme. Cell survival in the experimental group was similar to that in the control group, with no red color representing death observed in the experimental group, proving the biosafety of the nanoenzyme (Supplementary Fig. 7). To determine whether the nanoenzyme scavenges ROS in neuronal HT22 cells, intracellular ROS levels were examined using an ROS assay kit (2',7’-dichlorodihydrofluorescein diacetate [DCFH-DA]). Lipopolysaccharide (LPS) acted as an ROS-inducing drug, and intracellular ROS (green fluorescence) induced by LPS was observed in the LPS group (Fig. 3a), indicating that treatment with LPS elevated the ROS levels in cells^24^. Compared with the LPS group, Co_3_O_4_, Mn/Co_3_O_4_, and P/D@Mn/Co_3_O_4_ groups exhibited significantly lower intracellular ROS levels. Mn/Co_3_O_4_ and P/D@Mn/Co_3_O_4_ groups exhibited better ROS scavenging effects than the Co_3_O_4_ group, possibly due to the addition of Mn that enhanced the CAT-like catalytic activity of the nanoenzyme. Using flow cytometry, we found that P/D@Mn/Co_3_O_4_ effectively reduced the ROS levels in cells (Fig. 3b).

To further determine whether P/D@Mn/Co_3_O_4_ NPs prevent apoptosis by eliminating ROS, we performed an apoptosis detection assay in neuronal HT22 cells. In the positive control group treated with ROS (H_2_O_2_), the apoptosis rate reached 71.6%. After the addition of P/D@Mn/Co_3_O_4_ NPs, the apoptosis rate significantly decreased to 7.24% (Supplementary Fig. 8). To further demonstrate the alleviation of inflammation after the elimination of ROS, we measured the secretion of inflammatory factors in HT22 cells using an enzyme-linked immunosorbent assay. We found that the secretion of inflammatory factors, tumor necrosis factor-alpha, and interleukin-6 (IL-6) were significantly reduced in the P/D@Mn/Co_3_O_4_ group than in the LPS group (Fig. 3c, d). These results indicate that P/D@Mn/Co_3_O_4_ NPs alleviate inflammation by lowering ROS levels and reducing the tissue injury^25^. In summary, P/D@Mn/Co_3_O_4_ nanoenzyme exhibits enzyme-like catalytic properties to scavenge ·OH and reduces cell inflammatory response^26^.

### P/D@Mn/Co_3_O_4_ NPs affect mitophagy in the neuroinflammatory environment

Cellular experiments revealed that P/D@Mn/Co_3_O_4_ NPs effectively scavenged ROS in biological environments, prompting us to investigate whether P/D@Mn/Co_3_O_4_ NPs indirectly affected MMP and induced mitophagy in the process of scavenging ROS. First, we performed co-localization experiments to verify whether P/D@Mn/Co_3_O_4_ NPs can reach the mitochondria. P/D@Mn/Co_3_O_4_ was labeled with the fluorescent marker, Nile Red (NR), and co-incubated with HT22 cells for 12 h. Using confocal microscopy, we observed the colocalization (yellow spots) of mitochondria (green) and P/D@Mn/Co_3_O_4_ (red) and found that P/D@Mn/Co_3_O_4_ could reach the mitochondria (Fig. 3g). Next, to test whether P/D@Mn/Co_3_O_4_ induces mitophagy, we assessed MMP by incubating cells with P/D@Mn/Co_3_O_4_ and using the 5,5′,6,6′-tetrachloro-1,1′,3,3′-tetraethylbenzimidazolyl-carbocyanine iodide (JC-1) probe. JC-1 spontaneously aggregates in normal mitochondria, forming JC-1 aggregates at high membrane potentials and emitting red fluorescence. In apoptotic cells, JC-1 remains a monomer and exhibits green fluorescence due to MMP depolarization^27^. As shown in Fig. 3e, green fluorescence was observed in both the carbonyl cyanide-m-chlorophenylhydrazone (CCCP)-treated (positive control) and P/D@Mn/Co_3_O_4_ groups, indicating that both P/D@Mn/Co_3_O_4_ and CCCP induce MMP depolarization. Moreover, green fluorescence increased with the gradual increase in the concentration of P/D@Mn/Co_3_O_4_. Notably, 30 µg/mL P/D@Mn/Co_3_O_4_ was the most potent in inducing MMP depolarization.

As the reduction in MMP induces mitophagy, we used a mitochondrial kit to detect whether mitochondria underwent autophagy in this study. Mtphagy Dye, a reagent used to detect mitophagy in intact mitochondria, emits a faint red fluorescence^28^. Lyso Dye is used to label lysosomes and emits green fluorescence. During mitophagy, damaged mitochondria fuse with lysosomes, and Mtphagy and Lyso dyes emit strong red and green fluorescence, respectively. In the control group, the fluorescence was mainly green, indicating the absence of autophagy. In contrast, red and green fluorescence was observed in the CCCP-treated and P/D@Mn/Co_3_O_4_ groups, indicating the occurrence of mitophagy (Fig. 3f). In the experimental group treated with P/D@Mn/Co_3_O_4_ NPs, we observed a significant increase in the mRNA expression levels of phosphatase and tensin homolog-induced kinase 1 (PINK1) and sequestosome-1 (P62) along with a significant increase in the microtubule-associated protein 1A/1B-light chain 3 (LC3)-II to LC3-I ratio (Supplementary Fig. 9–11). PINK1, P62, and LC3 are essential proteins for mitophagy initiation^29^. In response to membrane depolarization, PINK1 accumulates in the outer mitochondrial membrane and recruits Parkin to ubiquitinate damaged mitochondria, promoting autophagy^30^. LC3 recruitment by the autophagosome assembly is a marker of autophagy^31^. Increased expression of these two genes further confirmed the occurrence of mitophagy and ubiquitination degradation of mitochondria.

Next, we explored the cause of mitophagy induced by P/D@Mn/Co_3_O_4_. We examined the ROS levels in the cell supernatant at different time points after P/D@Mn/Co_3_O_4_ treatment and found that ROS were largely eliminated within 30 min (Fig. 3h). In addition, we tested the activities of the respiratory chain complexes I−V (Supplementary Fig. 12). Experimental group exhibited significant abnormalities in the respiratory chain complex activities compared with the control group, indicating that the addition of P/D@Mn/Co_3_O_4_ interfered with the regular operation of the mitochondrial respiratory chain. We also examined the nicotinamide adenine dinucleotide (NAD^+^) levels in cell supernatants. NAD^+^ levels in the experimental group were significantly lower than those in the control group, indicating the depletion of H^+^ in the mitochondrial respiratory chain (Fig. 3i). Based on these results, we suggested a mechanism by which P/D@Mn/Co_3_O_4_ influences mitophagy in the neuroinflammatory environment. P/D@Mn/Co_3_O_4_ catalyzes hydrogen ions and ·OH to generate water and scavenges excess ROS while consuming large amounts of H^+^, thereby preventing neuronal cell apoptosis and maintaining MMP, which induces mitophagy and fundamentally reduces subsequent ROS production. Autophagy plays a protective role in neurons, and defects in autophagy have been reported in many neurodegenerative diseases^32,33^. Therefore, mitophagy-inducing effect favored the neuronal protective effect of P/D@Mn/Co_3_O_4_ NPs .

### Brain-targeting efficiency of P/D@Mn/Co_3_O_4_ NPs in vivo

**Fig. 4:**
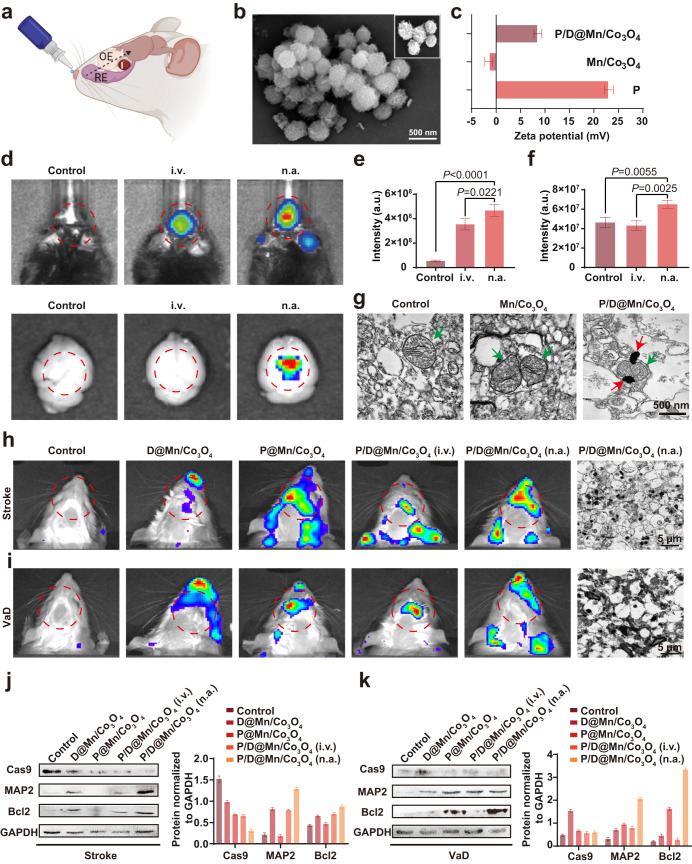
Nasal administration of P/D@Mn/Co_3_O_4_ and its effects on stroke and vascular dementia (VaD). **a** P/D@Mn/Co_3_O_4_ was administrated through a nasal spray and entered the damaged region of the brain via the olfactory pathway. OE, olfactory epithelium; RE, respiratory epithelium. **b** Scanning electron microscopy (SEM) image of P/D@Mn/Co_3_O_4_ NPs (Scale bar: 500 nm). **c** Zeta potential of Mn/Co_3_O_4_ NPs before and after coating. **d** Levels of P/D@Mn/Co_3_O_4_ reaching the brain after intravenous (i.v.) and nasal administration (n.a.). **e** Fluorescence quantification analysis for the fluorescence imaging of the head of rats shown in panel (**d**). **f** Fluorescence quantification analysis for the fluorescence imaging of the dissected brain of rats shown in panel (**d**). **g** TEM image of mitochondria in the inflammatory region of the mouse brain after establishing the disease model via embolization (Scale bar: 500 nm). Red arrows indicate P/D@Mn/Co_3_O_4_, and green arrows indicate mitochondria. **h** Brain-reaching efficiencies of NPs with different surface modifications in the stroke model, and TEM images of the brain injury sites (Scale bar: 5 µm). **i** Brain-reaching efficiencies of NPs with different surface modifications, and TEM images of the brain injury sites in the VaD model. Pink arrows indicate P/D@Mn/Co_3_O_4_ (Scale bar: 5 µm). **j** Western blots of caspase 9 (Cas9), microtubule-associated protein-2 (MAP2), and B-cell lymphoma-2 (Bcl2) levels in the stroke model. **k** Western blots of Cas9, MAP2, and Bcl2 levels in the VaD model.Data in (**c**), (**e**), and (**f**) are represented as the mean values ± SD. One-way ANOVA with Bonferroni post hoc test for pair-wise comparisons. *P* > 0.05 "not significant"; *P* < = 0.05 "significant", *P* < = 0.01 "very significant. *n* = 5 biologically independent samples in (**c**), (**e**), and (**f**). Representative images of four biologically independent samples from each group are shown in (**b**), (**d**), (**g**), (**h**), (**i**), (**j**), (**k**), and (**g**).

Cellular experiments demonstrated the excellent performance of P/D@Mn/Co_3_O_4_ NPs in eliminating ROS and promoting mitophapy in biological environments. Next, we verified its performance via animal experiments. We chose the nasal administration route to pass the drug through the trigeminal and olfactory pathways, bypassing the BBB and efficiently entering the brain (Fig. 4a). The surface of P/D@Mn/Co_3_O_4_ NPs was wrapped with an artificial cell membrane consisting of DOTAP and platelet membranes. Platelet membranes can target brain injury sites^34,35^. DOTAP increases the positive surface charge of P/D@Mn/Co_3_O_4_ NPs and confers it the ability to efficiently target mitochondria having a negative surface charge^36^. We observed Mn/Co_3_O_4_ and P/D@Mn/Co_3_O_4_ using scanning electron microscopy (SEM) and identified a thin film on the surface of P/D@Mn/Co_3_O_4_ NPs with a spherical morphology (Fig. 4b; Supplementary Fig. 13). Then, we investigated the zeta potential of P/D@Mn/Co_3_O_4_. Zeta potential changed from negative to positive, confirming the successful modification of the platelet membranes and DOTAP. Moreover, the positive surface potential of P/D@Mn/Co_3_O_4_ facilitated the targeting of abnormal mitochondria upon reaching the site of neuroinflammation (Fig. 4c).

Next, to demonstrate the superiority of nasal administration over other forms of administration, we performed nasal and injectable administration of NR-labeled P/D@Mn/Co_3_O_4_, followed by the use of an in vivo imaging system (IVIS) to detect the distribution of P/D@Mn/Co_3_O_4_ in mice 8 h after administration. The most robust fluorescence was observed in the nasal administration group (Fig. 4d), indicating that nasal administration is the preferred route for P/D@Mn/Co_3_O_4_ administration. These results were confirmed via fluorescence quantification analysis of the head and dissected brains of mice (Fig. 4e, f). The nasal administration of NPs directly introduces them into the brain via the olfactory pathway, bypassing BBB and enhancing its bioavailability in the brain^37^. To demonstrate that P/D@Mn/Co_3_O_4_ reached the neuroinflammatory site in mice and targeted the mitochondria, we sampled and sectioned the neuroinflammatory region in mice and observed the mitochondria and peri-mitochondrial region via TEM. We found that P/D@Mn/Co_3_O_4_ reached the neuroinflammatory region and targeted mitochondria for tight apposition, facilitating the indirect consumption of H^+^ by P/D@Mn/Co_3_O_4_ via its enzyme-like activity (Fig. 4g). Interestingly, we also detected the presence of autophagosomes, confirming the induction of mitophagy by P/D@Mn/Co_3_O_4_ (Supplementary Fig. 14).

To confirm the ability of platelet membranes and DOTAP to target inflammatory regions and mitochondria, we used an IVIS after the intranasal administration of different compositions of membrane-coated Mn/Co_3_O_4_. D@Mn/Co_3_O_4_ indicates Mn/Co_3_O_4_ wrapped with DOTAP only, P@Mn/Co_3_O_4_ indicates Mn/Co_3_O_4_ wrapped with platelet membranes only, and P/D@Mn/Co_3_O_4_ indicates Mn/Co_3_O_4_ wrapped with both platelet membranes and DOTAP. We found that the nasally administered P/D@Mn/Co_3_O_4_ group exhibited intense fluorescence in both the stroke and VaD models, indicating that P/D@Mn/Co_3_O_4_ NPs have good brain-targeting ability owing to the artificial cell membranes composed of platelet membranes and DOTAP (Fig. 4h, i). In the stroke model, caspase-9 levels were lower in the P/D@Mn/Co_3_O_4_ nasal administration group than in the control group (Fig. 4j, Supplementary Fig. 15a), indicating a decrease in apoptosis via the mitochondrial pathway. In addition, B-cell lymphoma-2 (Bcl2) and microtubule-associated protein-2 (MAP2) levels were increased in the P/D@Mn/Co_3_O_4_ nasal administration group, indicating a decrease in neuronal cell apoptosis and survival of a large number of neurons. In the VaD model, nasal administration of P/D@Mn/Co_3_O_4_ increased Bcl2 levels to some extent compared to those in the control group, indicating a decrease in neuronal apoptosis and a significant increase in MAP2 levels, favoring neuronal cell survival (Fig. 4k, Supplementary Fig. 15b).

### Therapeutic effects of P/D@Mn/Co_3_O_4_ NPs in the stroke model

**Fig. 5:**
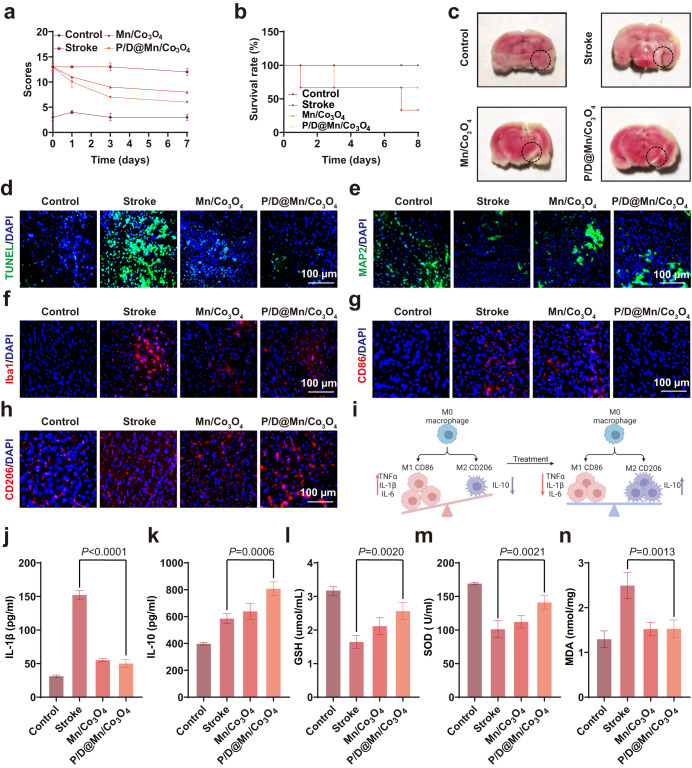
P/D@Mn/Co_3_O_4_ relieves disease symptoms in model rats. **a** Modified Neurological Severity Score (mNSS) test scores. **b** Survival efficiency of rats after different treatments. **c** Brain sections showing regions of stroke damage (stippled areas). **d**–**f** Representative fluorescence images of brain sections exhibiting neuronal cell apoptosis (terminal deoxynucleotidyl transferase-mediated dUTP nick-end labeling [TUNEL]) (e), number of neurons (MAP2) (f), and number of microglia (ionized calcium-binding adapter molecule 1 [Iba1]) (**g**) in stroke rats (Scale bar: 100 µm). **g** Immunofluorescent staining of M1 microglia in the brains of different treatment groups (Scale bar: 100 µm). **h** Immunofluorescent staining of M2 microglia in the brains of different treatment groups (Scale bar: 100 µm). **i** M1 microglia polarized to M2 microglia in the brains of apoplexy rats after P/D@Mn/Co_3_O_4_ treatment. **j** IL-1β level in the brain. **k** IL-10 level in the brain. **l** Glutathione (GSH) level in the brain. **m** Superoxide dismutase (SOD) level in the brain. **n** Malondialdehyde (MDA) level in the brain. Data in (**a**), (**j**), (**k**), (**l**), (**m**), and (**n**) are represented as the mean values ± SD. One-way ANOVA with Bonferroni post hoc test for pair-wise comparisons. *P* > 0.05 "not significant"; *P* < = 0.05 "significant", *P* < = 0.01 "very significant. *n* = 5 biologically independent samples in (**a**), (**j**), (**k**), (**l**), (**m**), and (**n**). Representative images of four biologically independent samples from each group are shown in (**c**), (**d**), (**e**), (**f**), (**g**), and (**h**).

After demonstrating the brain-targeting ability of P/D@Mn/Co_3_O_4_ NPs, we investigated their curative effects on cerebral ischemia. A rat stroke model was established using the bolus method to simulate acute cerebral ischemia-induced stroke (Supplementary Fig. 16). NPs were administered immediately after verifying successful modeling, followed by nasal administration four times once every 8 h. Subsequently, neurological scoring was performed. As shown in Fig. 5a, the modified Neurological Severity Scores (mNSSs) in the experimental group were significantly lower than those in the stroke-alone group (Supplementary Table. 1). In particular, stroke rats treated with P/D@Mn/Co_3_O_4_ exhibited ameliorated symptoms and improved mobility, approaching the level observed in the control group (normal rats), demonstrating the therapeutic potential of P/D@Mn/Co_3_O_4_. In addition, the survival rate was significantly higher in the P/D@Mn/Co_3_O_4_ group than in the stroke and Mn/Co_3_O_4_ groups, whereas the survival rate remained at 100% for eight weeks (Fig. 5b). Rat tissues were dissected and stained with 2,3,5-triphenyl-2H-tetrazolium chloride (TTC). The experimental group had less brain damage, which was close to the level observed in the control group (Fig. 5c). Brain tissue was sectioned and stained for terminal deoxynucleotidyl transferase-mediated dUTP nick-end labeling (TUNEL) assay. Green fluorescence in the experimental group was low compared with that in the stroke group, which was close to that of the control group, indicating an improvement in neuronal cell apoptosis (Fig. 5d). To further observe the neuronal condition, the neuronal status was evaluated via MAP2 immunostaining. Fluorescence intensity in the experimental group was significantly higher than that in the stroke group, indicating that P/D@Mn/Co_3_O_4_ NPs protected neuronal cells from ROS damage (Fig. 5e). We further observed the changes in microglia via ionized calcium-binding adapter molecule 1 (Iba1) immunostaining. We found that the number of microglia in the experimental group was significantly reduced compared to that in the stroke group, reaching a level similar to that in healthy control rats (Fig. 5f). Iba-1-positive microglia are positively associated with the neuroinflammatory response in stroke^38^. A reduction in the number of microglia may imply the attenuation of the inflammatory state in the brain. Therefore, we investigated the effect of P/D@Mn/Co_3_O_4_ on the inflammatory response in the ischemic brain after stroke.

Ischemia-reperfusion injury is often accompanied by inflammation, with microglia polarizing to M1 state and releasing large amounts of proinflammatory factors that mediate a robust inflammatory response and exacerbate the tissue injury. To detect brain inflammation, we used CD86 as a marker of M1 microglia and CD206 as a marker of M2 microglia. P/D@Mn/Co_3_O_4_ NPs decreased M1 and increased M2 polarization compared with that in the stroke group, suggesting that the microglia in the experimental group underwent conversion from M1 to M2 state, inhibiting the formation of an inflammatory environment (Fig. 5g–i). After treatment, levels of the pro-inflammatory factor, IL-1β, and anti-inflammatory factor, IL-10, were determined. IL-1β levels were significantly reduced, and IL-10 levels were significantly increased in the experimental group (Fig. 5j, k). These results indicate the alleviation of inflammation by P/D@Mn/Co_3_O_4_ owing to its anti-inflammatory effects and ability to regulate microglial polarization.

Under normal conditions, free radicals and antioxidants, such as glutathione (GSH) and superoxide dismutase (SOD), are in equilibrium with each other^39^. In ischemia-reperfusion injury, the production of free radicals increases and the activity of free radical-related enzymes decreases, resulting in a drastic increase in free radical levels in the body. Excess free radicals consume these balancing substances, further damaging cell membranes and producing large amounts of lipid peroxides, such as malondialdehyde (MDA), eventually causing cell death or necrosis. To demonstrate the overall condition of the brain, the brain tissue was homogenized and levels of GSH, MDA, SOD, and other substances were determined. GSH and SOD levels were significantly higher in the experimental group than in the stroke group (Fig. 5l, m). In contrast, MDA levels were significantly low, approaching the levels in the control group (Fig. 5n). Increased levels of GSH and SOD scavenge ROS and protect cells against oxidative insult^40^.

### Therapeutic effects of P/D@Mn/Co_3_O_4_ NPs in the VaD model

**Fig. 6:**
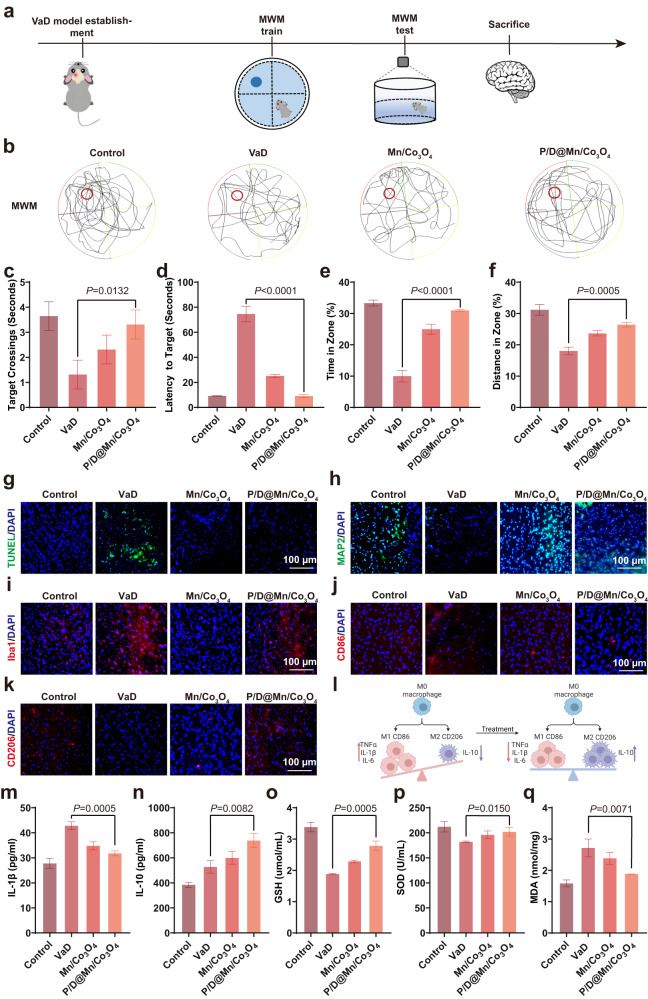
P/D@Mn/Co_3_O_4_ alleviates disease symptoms in the rat VaD model. **a** Schematic of the treatment process of rat VaD model with P/D@Mn/Co_3_O_4_. **b** Water maze test of rats in different treatment groups. **c** Incubation period in the water maze experiment. **d** Time taken to cross the platform in the water maze experiment. **e** Time spent in the target area in the water maze experiment. **f** Swimming distance of the target area in the water maze experiment. **g−i** Representative fluorescence images showing neuronal cell apoptosis (TUNEL) (**g**), number of neurons (MAP2) (**h**), and number of microglia (Iba1) (**i**) in VaD rats (Scale bar: 100 µm). **j** Immunofluorescence staining of M1 microglia in the brains of different treatment groups (Scale bar: 100 µm). **k** Immunofluorescence staining of M2 microglia in the brains of different treatment groups (Scale bar: 100 µm). **l** M1 microglia polarized to M2 microglia in the brains of apoplexy rats after P/D@Mn/Co_3_O_4_ treatment. **m** IL-1β level in the brain. **n** IL-10 level in the brain. **o** GSH level in the brain. **p** SOD level in the brain. **q** MDA level in the brain. Data in (**c**), (**d**), (**e**), (**f**), (**m**), (**n**), (**o**), (**p**), and (**q**) are represented as the mean values ± SD. One-way ANOVA with Bonferroni post hoc test for pair-wise comparisons. *P* > 0.05 "not significant"; *P* < = 0.05 "significant", *P* < = 0.01 "very significant. *n* = 5 biologically independent samples in (**c**), (**d**), (**e**), (**f**), (**m**), (**n**), (**o**), (**p**), and (**q**). Representative images of four biologically independent samples from each group are shown in (**b**), (**g**), (**h**), (**i**), (**j**), and (**k**).

To simulate chronic cerebral ischemia-induced dementia, a rat model of VaD was established via bilateral vein ligation (Supplementary Fig. 17). Then, the water maze experiment was performed after four days of training (Fig. 6a). The movement trajectory was captured and statistically analyzed during the test (Fig. 6b). The latency to reach the platform and the swimming distance were significantly shorter. The number of times through the platform and the time in the target region was significantly increased in the P/D@Mn/Co_3_O_4_-treated group compared to those in the VaD alone group (Fig. 6c–f). These phenomena indicated that the learning, memory, and spatial exploration abilities of VaD rats treated with P/D@Mn/Co_3_O_4_ were not significantly impaired, which provided further evidence of the validity of our therapy.

After demonstrating the treatment effects at the macroscopic level, we investigated the effects at the microscopic level. Frozen sections of rat brains were cut and stained with TUNEL, MAP2, and Iba1. The results showed that TUNEL fluorescence was significantly decreased (Fig. 6g), and MAP2 fluorescence was significantly increased in the P/D@Mn/Co_3_O_4_-treated group compared to that in the VaD alone group (Fig. 6h), indicating that our therapy can protect the neural cells from apoptosis. Moreover, the microglia were significantly activated (Fig. 6i) and polarized to M1 (Fig. 6j) and released large amounts of the pro-inflammatory factor IL-1β in the VaD group. In P/D@Mn/Co_3_O_4_ treatment, VaD rats exhibited fewer microglia, which shifted from the pro-inflammatory M1 to the anti-inflammatory M2 phenotype under the action of P/D@Mn/Co_3_O_4_ NPs (Fig. 6k, l). In addition, the experimental group showed effective inhibition of IL-1β and a significant increase in the levels of the anti-inflammatory factor, IL-10 (Fig. 6m, n). We further investigated the underlying protective mechanisms of NPs. Levels of antioxidants (GSH and SOD) in the P/D@Mn/Co_3_O_4_-treated group were significantly higher than those in the VaD alone group (Fig. 6o, p). In contrast, the oxidation product (MDA) level was significantly lower in the P/D@Mn/Co_3_O_4_-treated group than in the VaD alone group (Fig. 6q). Therefore, P/D@Mn/Co_3_O_4_ NPs delay the intense inflammatory response and reduce oxidative injury, thereby exerting potent therapeutic effects on VaD.

To evaluate the residence time of P/D@Mn/Co_3_O_4_ NPs in vivo after injection, the olfactory bulb, liver, and kidney tissues injected with P/D@Mn/Co_3_O_4_ NPs for 1, 4, 7, and 14 d were analyzed via ICP-MS to observe the changes in Co. Mn/Co_3_O_4_ was still present in the olfactory bulb tissues on day 7 but was completely eliminated from the body by day 14 (Supplementary Fig. 18).

## Discussion

In this study, we developed an inhalation nanotherapeutic, P/D@Mn/Co_3_O_4_, to treat ICVD. The nasal administration of P/D@Mn/Co_3_O_4_ offered several advantages over intravenous administration. First, it minimized the negative effects on the liver, kidneys, and other organs while bypassing BBB through the trigeminal nerve and olfactory pathway, thereby increasing the levels of nanotherapeutic agents reaching the brain lesions. Second, the inclusion of platelet membranes enhanced the targeting of inflammation by P/D@Mn/Co_3_O_4_. We confirmed the efficiency of this nanotherapeutic in targeting inflammation in stroke and VaD models using in vivo imaging and TEM. Third, a positive charge on the surface of P/D@Mn/Co_3_O_4_ induced by DOTAP increased its accumulation around the mitochondria. Moreover, the enzyme-like activity of P/D@Mn/Co_3_O_4_ facilitated ROS scavenging, decreased oxidative stress, and prevented neuronal apoptosis. Its CAT-like activity scavenged intracellular ROS, thereby reducing the supply of H^+^ to the mitochondrial matrix and inducing mitophagy. In conclusion, the nanotherapeutic developed in this study can effectively treat ICVD by decreasing ROS production and preventing chronic and acute ICVD relapse.

## Methods

### Materials

Cobalt nitrate hexahydrate (Co(NO_3_)_3_·6H_2_O), glucose, and manganese chloride tetrachloride (MnCl_4_·4H_2_O) were purchased from Shanghai Aladdin Chemical Reagent Co., Ltd. Calcein-AM/PI live/dead cell double staining kit, Annexin V-FITC/PI apoptosis detection kit and DCFH-DA were obtained from Beijing Solarbio Science & Technology Co., Ltd. Caspase-9 (C9) Mouse mAb #9508 (1:1000), MAP2 (D5G1) XP® Rabbit mAb #8707 (1:1000), GAPDH (D16H11) XP® Rabbit mAb #5174 (1:1000), purchased from Cell Signaling Technology. Bcl-2 Monoclonal Antibody (10C4) (1: 1000) purchased from Thermo Fisher Scientific. Goat Anti-Rabbit IgG H&L / HRP antibody (bs-0295G-HRP) (1:10,000), Goat Anti-Mouse IgG H&L / HRP antibody (bs-0296G-HRP) (1:10,000), purchased from Bioss.

### Cell lines

Cell lines were obtained from Shanghai Jin Yuan Biotechnology Co.

### Animals

SD rats, weight: 200–250 g, purchased from Beijing HFK Bioscience Co., Ltd. C57BL/6 mice, weight: 18-20 g, purchased from Beijing Vital River Laboratory Animal Technology Co., Ltd. The Animal Ethics Committee of Tianjin University approved all animal experiments. To perform euthanasia of SD rats and C57BL/6 mice, the cervical dislocation method was used. Prior to euthanasia, each rat was anesthetized correctly to minimize any possible discomfort and pain. First, the rats were placed on a suitable bench to ensure the surrounding environment’s safety. The head is then stabilized, and gradually increasing forces are applied to trigger a break and dislocation between the first and second cervical vertebrae. Doing so quickly cuts the nerve and blood supply, causing the rat to lose consciousness and die rapidly.

### Synthesis of Co_3_O_4_ NPs

In this study, Co(NO_3_)_3_·6H_2_O (1 mmol) and glucose (1 mmol) were added to isopropanol (40 mL) and stirred for 30 min at room temperature. Then, 10 mL of glycerol was added, stirring the mixture for 1 h. The suspension was then subjected to a thermal reaction at 180 °C for 6 h. The final product was collected, washed with deionized water, and dried overnight under a vacuum at 60 °C.

### Synthesis of Mn/Co_3_O_4_ NPs

In this study, a mixture of Co(NO_3_)_3_·6H_2_O (1 mmol), MnCl_4_·4H_2_O (0.1 mmol), and glucose was prepared by stirring the components in 40 mL of isopropanol for 30 min at room temperature. After adding 10 mL of glycerol and further stirring for 1 h, the suspension was subjected to a hydrothermal reaction at 180 °C for 6 h. The product was then collected, washed with deionized water, and dried under a vacuum overnight at 60 °C.

### Synthesis of P/D@Mn/Co_3_O_4_ NPs

Whole blood was collected from 5- to 6-week-old male C57BL/6 mice (Beijing Vital River Laboratory Animal Technology Co., Ltd.) and centrifuged at 100 × g for 20 min to obtain platelet-rich plasma. After repeated centrifugation at 3000 × g for 20 min, platelet fractions were collected, washed with phosphate-buffered saline (PBS), and centrifuged centrifugation to collect the platelets. The prepared platelets were counted using a flow cytometer, and the number of platelets per tube was controlled at (2–5) × 10^7^. Next, 1 mL of membrane protein extraction reagent was added to the suspended platelets with phenylmethylsulfonyl fluoride. After ice bathing for 10–15 min, the suspension was centrifuged at 700 × g for 10 min at 4 °C, and the supernatant was collected. The supernatant was placed into a new centrifuge tube, extracted without touching the sediment, and centrifuged at 14,000 × g for 30 min at 4 °C to precipitate the platelet membrane fragments. After centrifugation, the platelet membrane fragments were weighed and mixed with DOTAP at a ratio (w/w) of 2:1. Subsequently, Mn/Co_3_O_4_ NPs were added to the mixture in a 1:5 ratio (membrane: Mn/Co_3_O_4_ NPs), stirred thoroughly, and squeezed using an extruder. After centrifugation at 8000 × g, P/D@Mn/Co_3_O_4_ was recovered and stored at 4 °C.

### Characterization

High-resolution TEM (Tecnai G2 F20, FEI) was used to observe the morphology and perform element mapping at an operating voltage of 200 kV. At room temperature, the particle size and zeta potential were determined via dynamic light scattering (Nano-Zetasizer ZS90, Malvern, UK). XPS was performed using an ESCALAB-250Xi system (Thermal Scientific, US). SEM photographs were recorded using a field-emission Magellan 400 microscope (FEI Company, US). X-ray diffraction was performed using a MiniFlex 600 diffractometer (Rigaku, Japan). Flow cytometry data were obtained using the CytoFLEX LX instrument.

### Cell culture and cytotoxicity experiments

HT22 cells were cultured in Dulbecco’s modified Eagle’s medium containing 10% fetal bovine serum and 1% penicillin/streptomycin. Cells were incubated with different concentrations of P/D@Mn/Co_3_O_4_ (0, 10, 20, 30, 40, 50, 60, 70, 80, 90, and 100 μg/mL; *n* = 3) in a 96-well plate for 24 h. Cytotoxicity was evaluated colorimetrically using the CCK-8 assay at 450 nm.

### VaD model establishment and treatment

The Animal Ethics Committee of Tianjin University approved all animal experiments. SD rats (weight: 200–250 g) were fasted for 12 h and injected intraperitoneally with 10% chloric acid hydrate (300 mL/kg). After anesthesia, rats were placed supine on the operating table. The common carotid artery was searched, and the subcutaneous tissue and fascia were bluntly dissected to avoid damage to the vagus nerve. After dissecting the bilateral common carotid arteries, a surgical thread was passed through the common carotid artery. A surgical thread was used to ligate the common carotid artery at the proximal end. A surgical thread was also used to ligate the carotid artery’s distal end, and the carotid artery was clipped. After ligation, the wound was flushed with saline and the muscle and skin were sutured. Penicillin was administered to the wound to prevent postoperative infections. After successful modeling, nasal administration of 100 μL of P/D@Mn/Co_3_O_4_ solution (80 μg/mL) was performed 1, 2, and 3 d after surgery.

### Stroke model establishment and treatment

SD rats (weight: 200–250 g) were fasted for 12 h and intraperitoneally injected with 10% chloric acid hydrate (300 mL/kg). After anesthesia, rats were placed in a supine position on the operating table. Blunt dissection of the sternocleidomastoid, brachialis, and sternocleidomastoid muscles was performed. Right, external, and internal carotid arteries were dissected. Care was taken to avoid any damage to the vagal nerve. Carotid and proximal carotid arteries were dissected. A small incision was made in the carotid artery, and a wire plug was inserted from the carotid bifurcation through the carotid artery into the middle cerebral artery. The wire plug was inserted to a depth of approximately 18–20 mm and secured to the middle artery using sutures to prevent slippage. The wounds were closed by suturing the skin. After establishing the ischemia-reperfusion model, the wire plug was slowly withdrawn from the common carotid artery to restore blood flow to the middle cerebral artery. After successful post-awake test modeling, group dosing was performed, followed by nasal dosing once every 8 h for a total of four doses of 100 μL P/D@Mn/Co_3_O_4_ solution (80 μg/mL).

### Determination of cerebral infarction

After collecting femoral artery blood, the skull was immediately opened. The whole brain was stripped, harvested, and placed at −20 °C for 15 min. The olfactory bulb and cerebellum were removed, and the remaining brain tissue was placed in a unique rat brain groove. The coronal plane was evenly cut into 2-mm thick brain slices (*n* = 5). Then, the brain slices were mixed with 10 mL PBS containing 1% TTC and bathed in water at 37 °C for 30 turns every 35 min.

### Apoptosis/necrosis assay

HT22 cells were seeded into a 96-well plate for 12 h to let them adhere, and then a certain amount of H_2_O_2_ was added to the medium. After 6 h culture, the medium was replaced with fresh medium containing P/D@Mn/Co_3_O_4_, and cells were further incubated for 12 h. Cells were washed with PBS and stained with V-FITC and PI in the dark, followed by washing and analysis by flow cytometry.

### Detection of ROS elimination from cells

HT22 cells were incubated in 96-well plates for 12 h. LPS was added and incubated for 6 h. Then the medium was replaced with fresh medium containing P/D@Mn/Co_3_O_4_ and further incubated for 12 h. Cells were washed with PBS and pretreated with DCFH-DA. Cells were incubated for another 30 min. Finally, cells were observed under a fluorescent microscope.

### Mitophagy study

HT22 cells were cultured in DMEM medium containing 10% fetal bovine serum and 1% penicillin-streptomycin and grown in a humidified incubator (5% CO_2_, 37 °C). Fresh medium containing P/D@Mn/Co_3_O_4_ was added. After 12 h incubation, the medium was discarded, cells were washed twice with serum-free medium, and an appropriate 100 nmol/L Mtphagy Dye working solution was added and incubated for 30 min at 37 °C. The supernatant was discarded, and cells were incubated with 1 μmol/L Lyso dye working solution for 30 min at 37 °C. The supernatant was discarded, the cells were washed twice with serum-free medium, and the cells were observed under a fluorescent microscope.

### Detection of mitochondrial membrane potential

HT22 cells were cultured in DMEM medium containing 10% fetal bovine serum and 1% penicillin-streptomycin and grown in a humidified incubator (5% CO_2_, 37 °C). Media containing P/D@Mn/Co_3_O_4_ was added. After 24 h of incubation, cells were incubated with 1 mg/L of JC-1 probe for 20 min at 37 °C. Cells were washed twice with cold PBS and observed under a fluorescent microscope.

### Training of the water maze

At the midpoints of four different quadrants, the rats were placed head down on the pool wall and allowed to swim freely in the water. If the rats found a platform and climbed on it within 60 s, they were allowed to stay on it for 30 s to reinforce the memory effect. If the rats did not find a platform within 60 s, the experimenter used the apparatus to lead them to the platform and allowed them to stay on it for 30 s to reinforce the memory effect. The above movement trajectory was recorded in real time with a camera recording system directly above the pool. At the end of the 5-day training experiment, the platform was removed, the rat was made to face the wall, and then was put down in the opposite quadrant of the original platform quadrant and let swim freely in the water for 90 s. Rat movement was recorded with the on-top real-time video recording system.

### Small animals live to image

Nile Red-labeled P/D@Mn/Co_3_O_4_ was used. The 1% Nile Red lipid dye was mixed with 50 µg/mL of P/D@Mn/Co_3_O_4_ and incubated for 30 min. 100 μL of Nile Red-labeled P/D@Mn/Co_3_O_4_ was administered intranasally to each group of mice. After 4 h, the mice were placed in a live small animal imaging device, and the fluorescence emission area was recorded. Excitation wavelength: 530 nm; emission wavelength: 630 nm.

### Frozen section and staining

At the end of the experiment, mice were executed by cervical dislocation, and the brains were harvested and fixed in 4% paraformaldehyde at 4 °C for 24 h. After fixation, the brains were continuously dehydrated with 20% and 30% sucrose solutions until they sank to the bottom of the liquid. After fixation with the embedding medium, the brains were immediately frozen and stored in liquid nitrogen at −80 °C. After freezing the samples, frozen 12–25 μm sections were cut with cryotome. Before staining, the sections were fixed with paraformaldehyde at 4 °C for 10–20 min, rinsed twice with PBS for 5 min each, and inactivated endogenous peroxidase with 3% H_2_O_2_ for 20 min avoiding light; sections were rinsed twice with PBS for 5 min each. Sections were incubated antibody at 37 °C for 1 h and then were washed twice with PBS for 5 min. Sections were incubated with DAPI at 37 °C for 40 min and washed twice with PBS each for 5 min.

### Determination of GSH-PX, SOD, and MDA

GSH-PX is a kind of catalytic ammonia peroxide widely existing in the body and was used to determine GSH-PX activity. The serum levels of SOD and MDA were determined according to the kit’s instructions.

### Reverse Transcription PCR (RT-PCR)

The RNA was acquired from the striatum with the TRIzol reagent. RNA concentrations were then evaluated with NanoDrop 2000 and utilized to combine the complementary DNA with a RevertAid First Strand cDNA synthesis kit (Thermo Fisher Scientific, MA, USA). Next, RT-PCR was performed with IL-10, IL-1β, β-actin, PINK1, LC3-I, LC3-II, and P62 primers on a Bio-Rad, CFX96 touch real-time system with gene PCR master mix (Thermo Fisher Scientific, MA, USA). Primers are listed below.

**Table:** 

IL-10-F	GCTCTTACTGACTGGCATGAG
IL-10-R	CGCAGCTCTAGGAGCATGTG
IL-1β-F	TGCCACCTTTTGACAGTGATG
IL-1β-R	AAGGTCCACGGGAAAGACAC
β-actin-F	GCTCTCATCACAGACGGGTT
β-actin-R	CTTCCTGCTGCCTTCACTCA
PINK1-F	TTC TTC CGC CAG TCG GTA G
PINK1-R	CTG CTT CTC CTC GAT CAG CC
LC3-I-F	GAC CGC TGT AAG GAG GTG C
LC3-I-R	CTT GAC CAA CTC GCT CAT GTT A
LC3-II-F	TTA TAG AGC GAT ACA AGG GGG AG
LC3-II-R	CGC CGT CTG ATT ATC TTG ATG AG
P62-F	AGG ATG GGG ACT TGG TTG C
P62-R	TCA CAG ATC ACA TTG GGG TGC

The relative mRNA expression of each group was compared utilizing the 2^−ΔΔCt^ method.

### Annexin V-FITC&PI apoptosis assay (flow cytometry)

The HT22 cells will be cultured and it is important to centrifuge each reagent before opening to ensure that the liquid is collected from the wall and mouth of the tube to avoid any loss. The 4× Binding Buffer should be diluted to 1× Binding Buffer with distilled water, and the collected cells should be gently washed with PBS and shaken. To resuspend the cells, 195 μL of 1× Binding Buffer should be added, and the cell density should be adjusted to 2–5 × 10^5^ cells/ml. Then, 5 μL of Annexin V-FITC should be added to 195 μL of cell resuspension, and the mixture should be protected from light, mixed well, and incubated at room temperature for 10–15 min. Following this, the cells should be washed with 200 μL of 1X Binding Buffer, then centrifuged at 1000 rpm for 2–5 min, and the supernatant discarded. To perform the flow assay, the cells should be resuspended in 190 μL of 1X Binding Buffer and 10 μL of Propidium Iodide should be added. It is essential to perform the flow assay within 4 h to prevent fluorescence decay.

### Statistical analysis

All experimental results are expressed as the mean ± standard deviation. The significance of group differences for normally distributed data was assessed using one-way analysis of variance followed by Tukey’s test. Statistical significance was set at *P* < 0.05. Other data were analyzed using GraphPad Prism 8.0.2 software.

### Supplementary information

supplementary materials

nr-reporting-summary

## Data Availability

All data needed to evaluate or reproduce the conclusions in the paper are present in the paper. The data supporting this study’s findings are available from the corresponding author.
