# Supplementary material for: Engineered inhaled nanocatalytic therapy for ischemic cerebrovascular disease by inducing autophagy of abnormal mitochondria
Source: NPJ Regen Med. 2023 Aug 11;8:44. doi: 10.1038/s41536-023-00315-1 (PMC10421937; doi:10.1038/s41536-023-00315-1)
Supplement: Supplementary file 1 — supplementary materials [file 41536_2023_315_MOESM1_ESM.pdf]

Supporting Information

Engineered inhaled nanocatalytic therapy for ischemic cerebrovascular disease by inducing autophagy of abnormal mitochondria

Deping Wang [1,2,‡], Bowen Li [1,‡], Shuchao Wang [1], Yingjian Hao [2], Hua Wang[3], Wei Sun [1], Jimin Cao [2,*], Xin Zhou [2,*] and Bin Zheng[1,*]

[1] Academy of Medical Engineering and Translational Medicine, Tianjin Key Laboratory of Brain Science and Neural Engineering, Xincheng Hospital of Tianjin University, Tianjin University, Tianjin 300072, China.

[2] Key Laboratory of Cellular Physiology, Ministry of Education, and the Department of Physiology, Shanxi Medical University, Taiyuan, 030001, China.

[3] School of Pharmaceutical Science and Technology, Tianjin University, 92 Weijin Road, Nankai District, Tianjin 300072, China.

[‡]These authors contributed equally to this work.

E-mail: caojimin@sxmu.edu.cn (JC); xzhou@sxmu.edu.cn (XZ);

binzheng@tju.edu.cn (BZ)

TABLE

Supplementary Table. 1. mNSS score of modified neural function defect in rats. Neurobehavioral scoring was performed 24h after modeling, and the scoring criteria are shown in the table below.

**Exercise test**	
**Tendence test**	3
The forelimb flexion	1
The hind limb flexion	1
The head deviates from the vertical axis> 100 within the 30s	1
**Place rats on the floor (normal =0; maximum =3)**	3
Walk normally	0
Can't walk straight	1
Turn to the light paraplegic side	2
Pour to the light paraplegic side	3
**Sensory testing**	2
Place test (visual and tactile test)	1
Onoceptive test (deep feeling, pressing the edge of the table to stimulate limb muscles)	1
**Balance beam test (normal value =0; maximum value =6)**	6
Stable balance posture	0

Grasp the edge of the balance beam	1
Hold the balance beam tightly, and one body falls from the balance beam	2
Hold the balance beam tightly, and the two limbs drop from or rotate on the balance beam (> 60 seconds)	3
Attempting to balance on the balance beam but drop (> 40 seconds)	4
Attempt to balance on the balance beam but drop (> 20 seconds)	5
Drop; not tried to balance on balance beam (<20 seconds)	6
**Loss of the reflex and abnormal movement**	4
Eicle reflex (shake head in contact with external ear canal)	1
Corneal reflex (blink when lightly touched with cotton silk)	1
Panic reflex (motor response to noise from bullet cardboard)	1
**Epilepsy, myoclonus, and dystonia**	1
**The highest score is 18**	

FIGURES

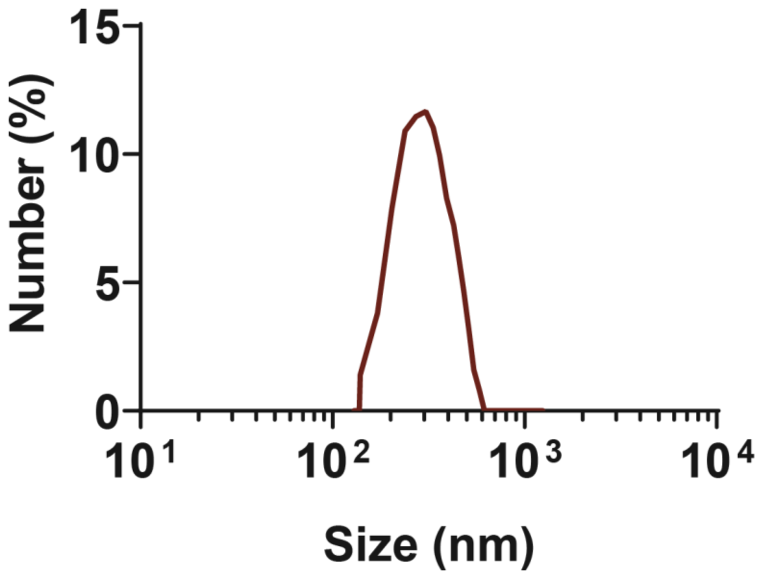

Supplementary Figure. 1. DLS analysis for the particle size of Mn/Co_3O_4 NPs.

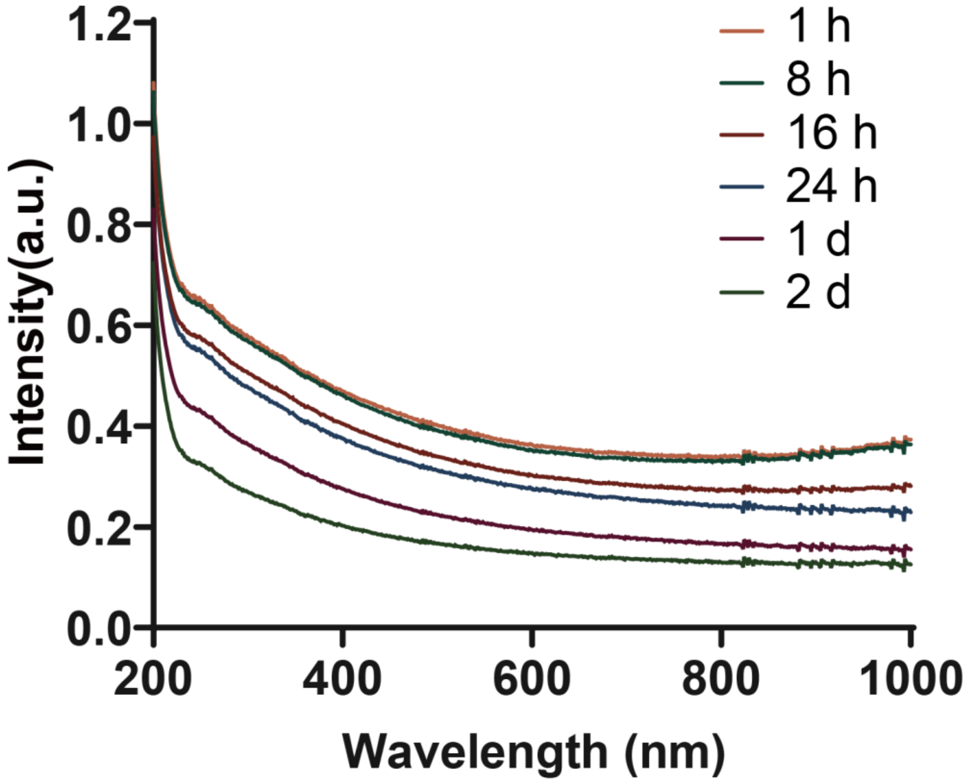

Supplementary Figure. 2. UV absorbance of Mn/Co_3O_4 NPs at different times.

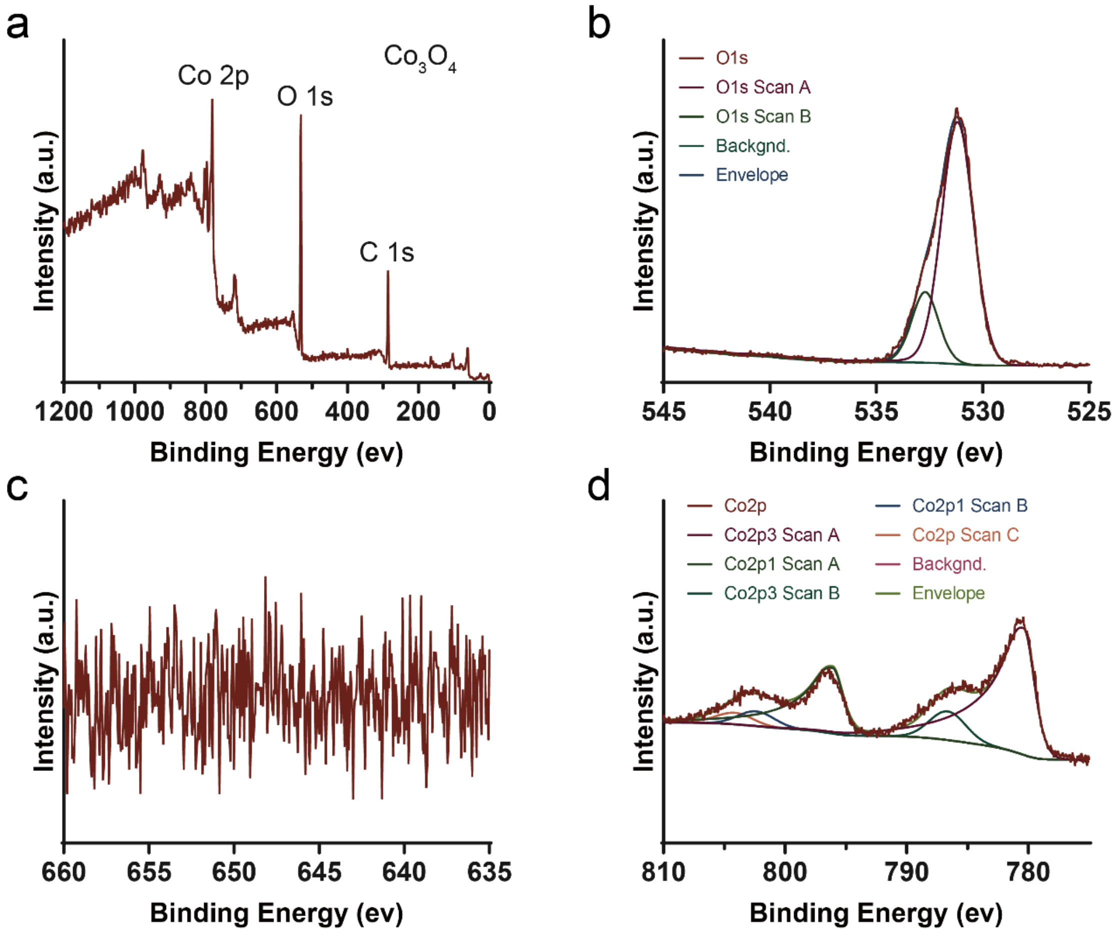

Supplementary Figure. 3. (a) XPS spectra of Co_3O_4. (b) XPS spectra of O 1s for Co_3O_4. (c) XPS spectra of Mn 2p for Mn/Co_3O_4. (d) XPS spectra of Co 2p for Mn/Co_3O_4.

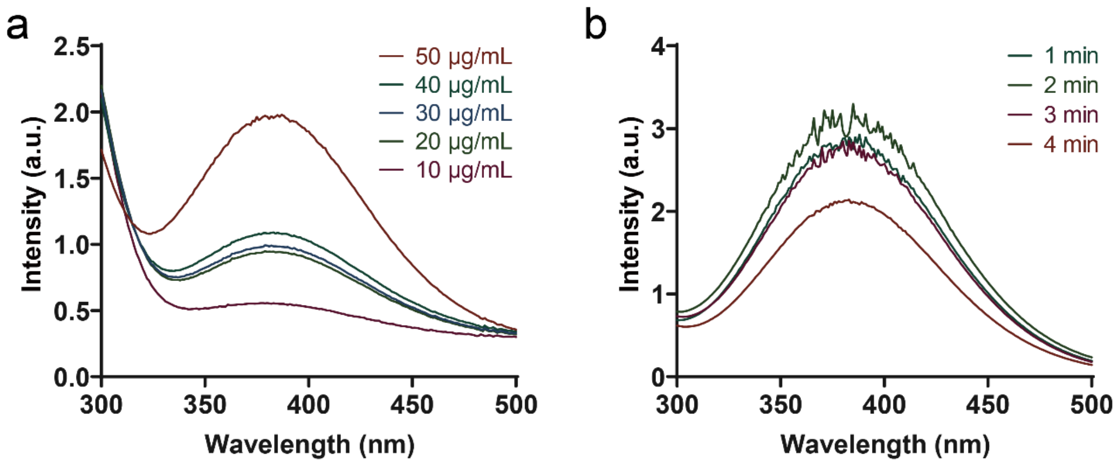

Supplementary Figure. 4. The concentration- and time-dependent CAT-like catalytic properties of Mn/Co_3O_4 NPs for the decomposition of H_2O_2.

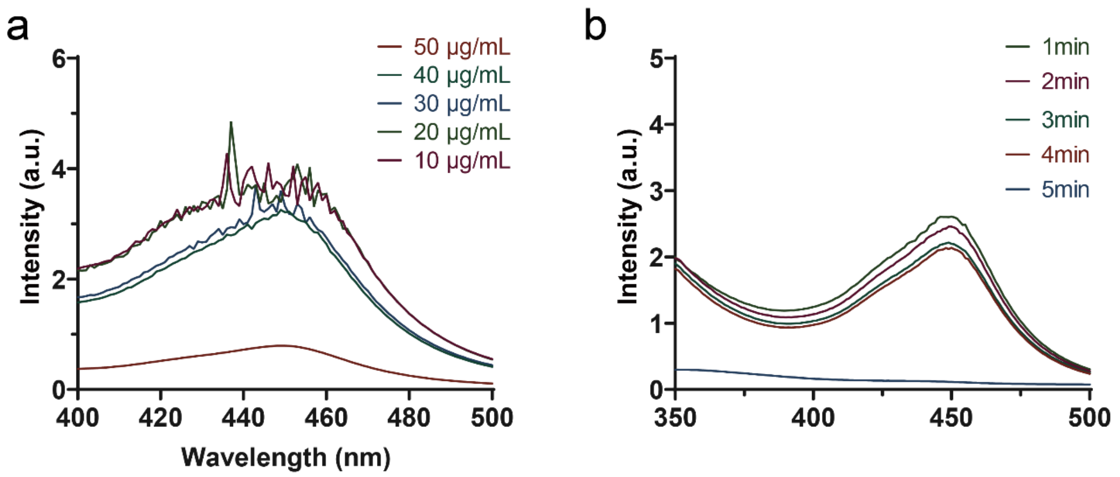

Supplementary Figure. 5. The concentration- and time-dependent catalytic properties of Mn/Co_3O_4 NPs for eliminating ·OH.

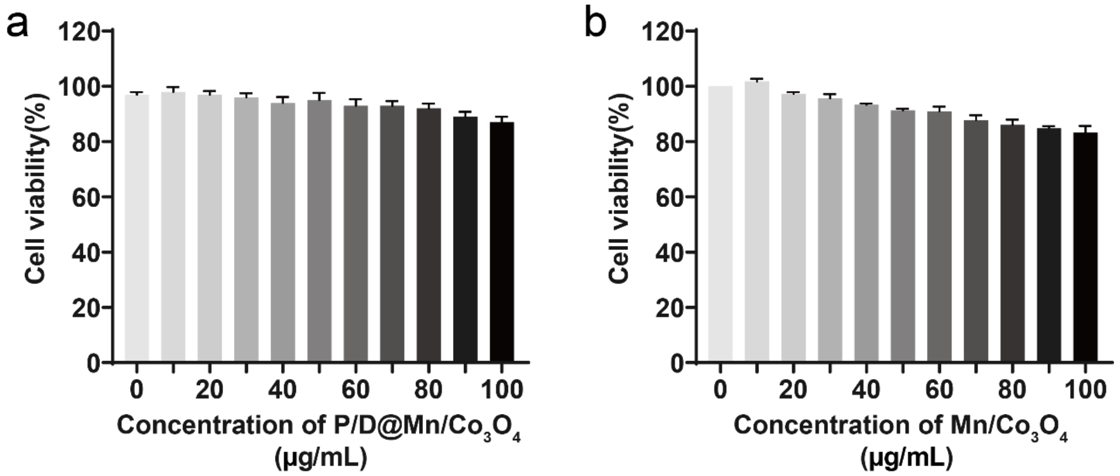

Supplementary Figure. 6. Biocompatibility test for $P/D@Mn/Co_3O_4$ NPs. (a) HT22 cells were incubated with different concentrations of $P/D@Mn/Co_3O_4$ for 24 h, and cell survival rate was detected using CCK-8 kits. (b) HT22 cells were incubated with different concentrations of Mn/Co_3O_4 for 24 h, and the cell survival rate was detected using CCK-8 kits.

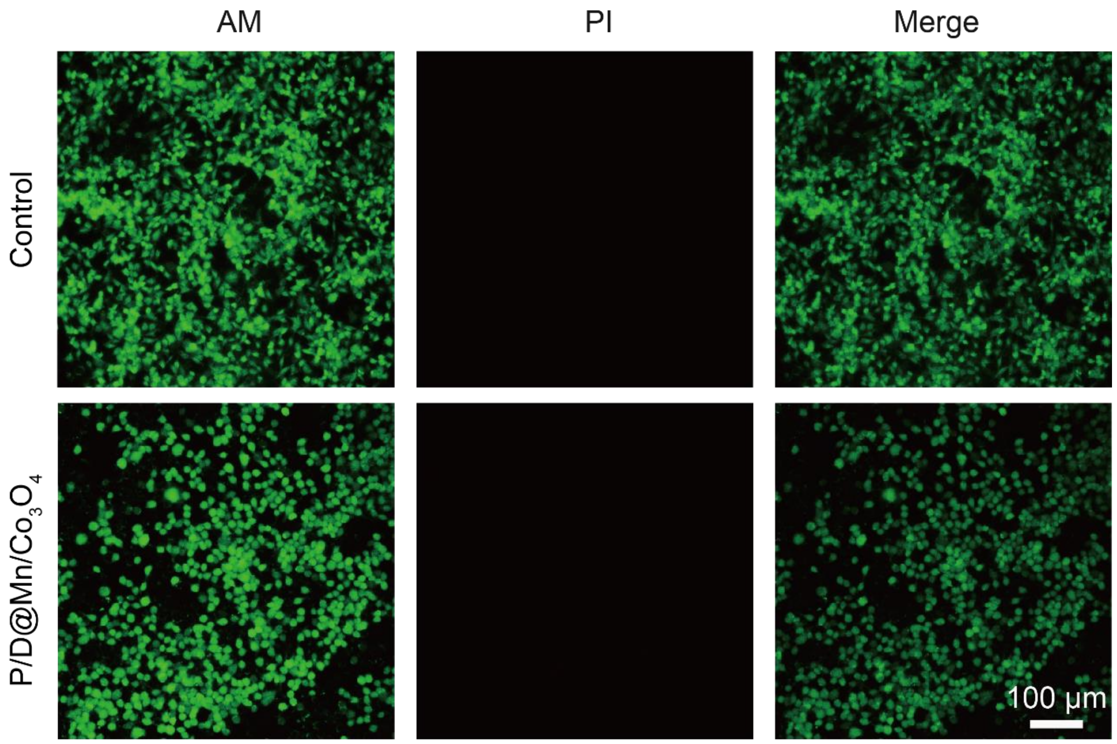

Supplementary Figure. 7. Biocompatibility of P/D@Mn/Co_3O_4 NPs assayed by calcein-AM/PI staining. HT22 cells were incubated with 60 μg/mL P/D@Mn/Co_3O_4 for 24 h, and the number of dead and living cells was detected to analyze the biocompatibility via calcein-AM/PI staining. Scale bar: 100 μm.

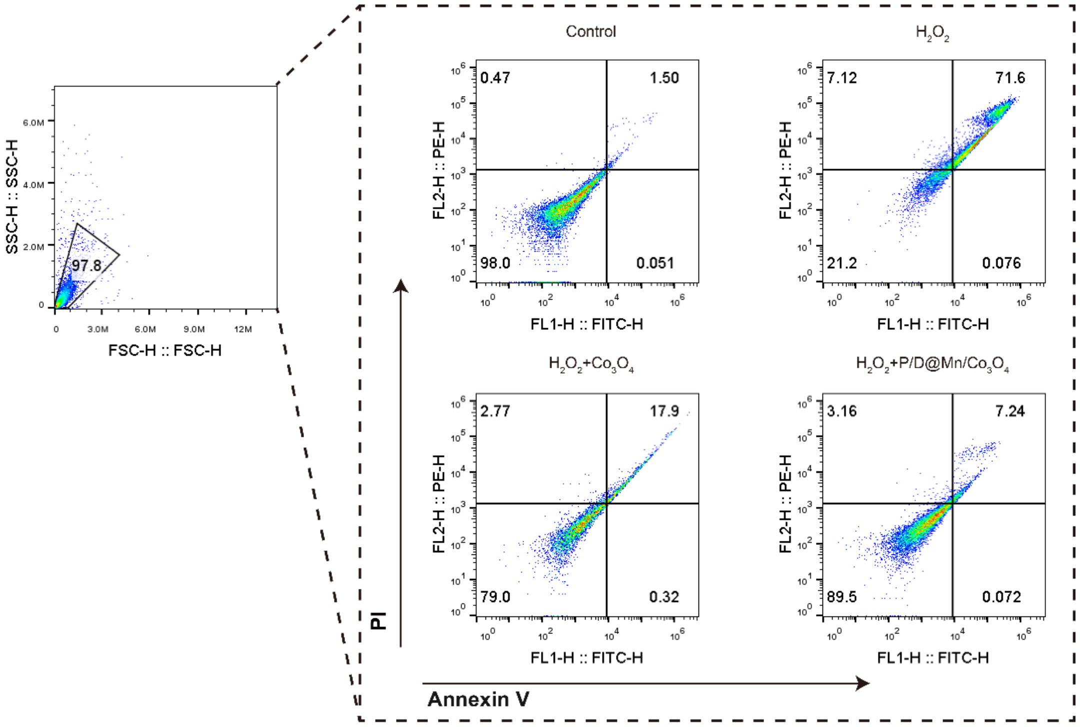

Supplementary Figure. 8. Flow cytometry analysis of HT22 cells showing the numbers of apoptotic cells with different treatments, including the gating strategy.

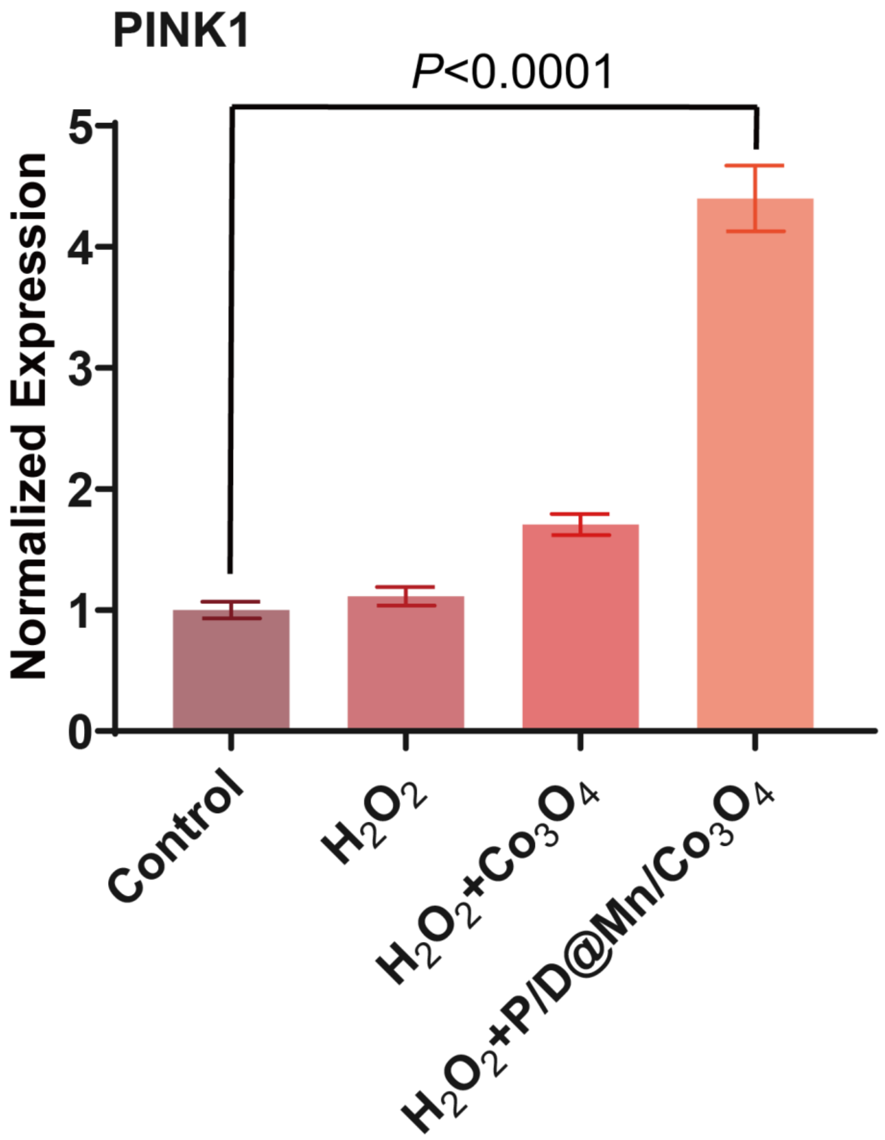

Supplementary Figure. 9. RT-PCR assay to detect the mRNA expression level of mitochondrial autophagy-related factor PINK1.

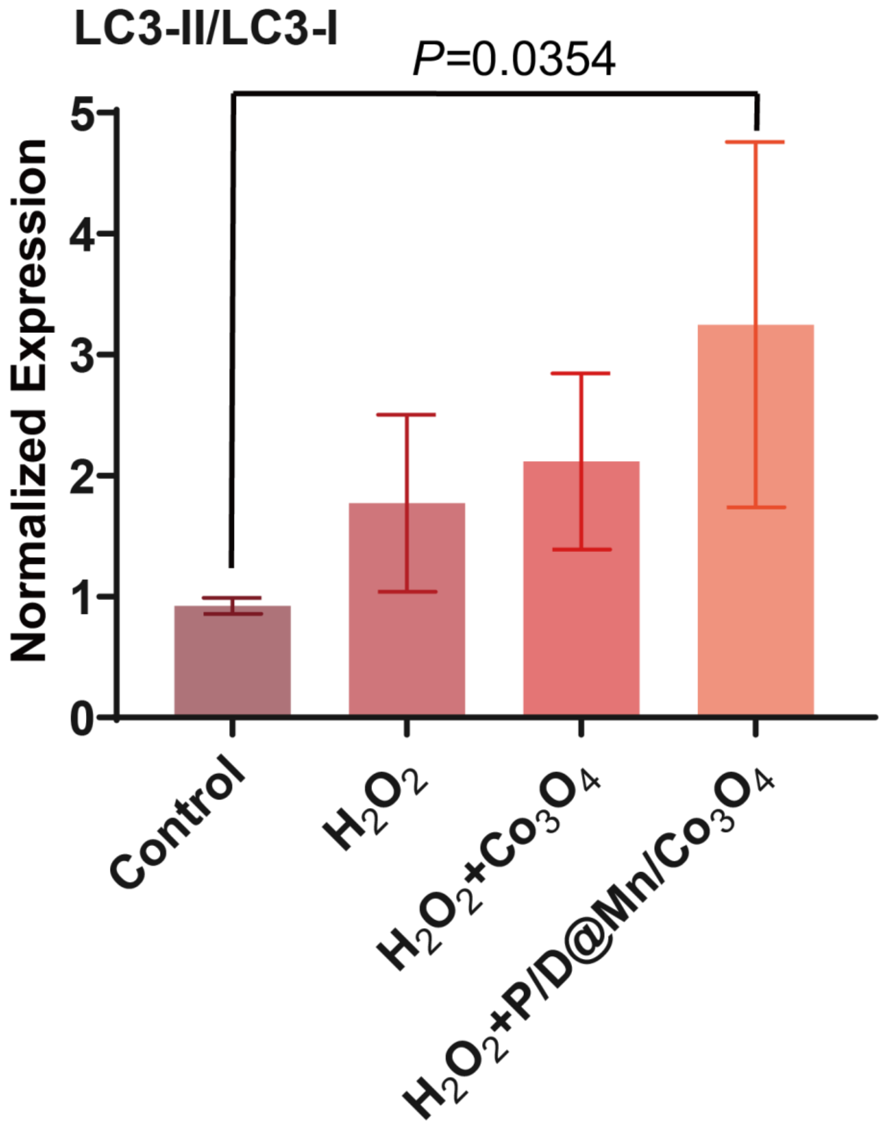

Supplementary Figure. 10. RT-PCR assay to detect the mRNA expression level of mitochondrial autophagy-related factor LC3-II/LC3-I.

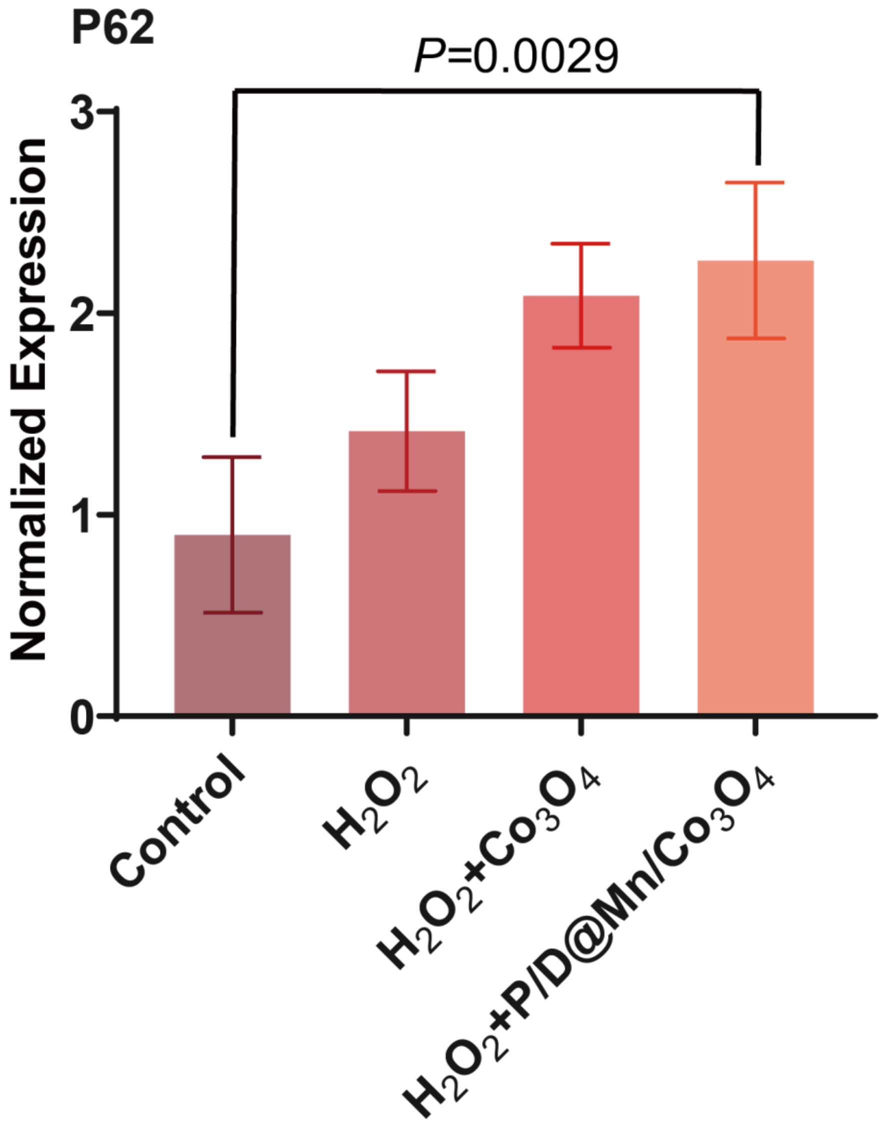

Supplementary Figure. 11. RT-PCR assay to detect the mRNA expression level of mitochondrial autophagy-related factor P62.

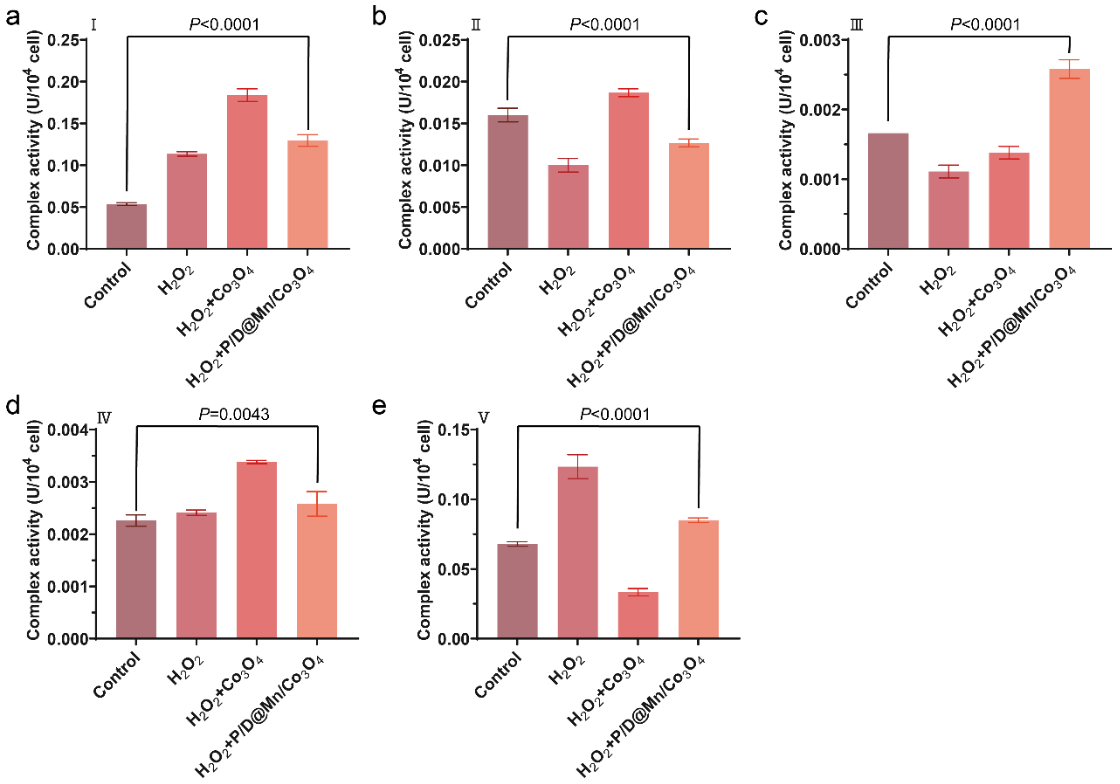

Supplementary Figure. 12. Effects of different treatments on the activities of mitochondrial respiratory chain complexes I–V examined with a Mitochondrial Respiratory Chain Complexes Activity Assay Kits.

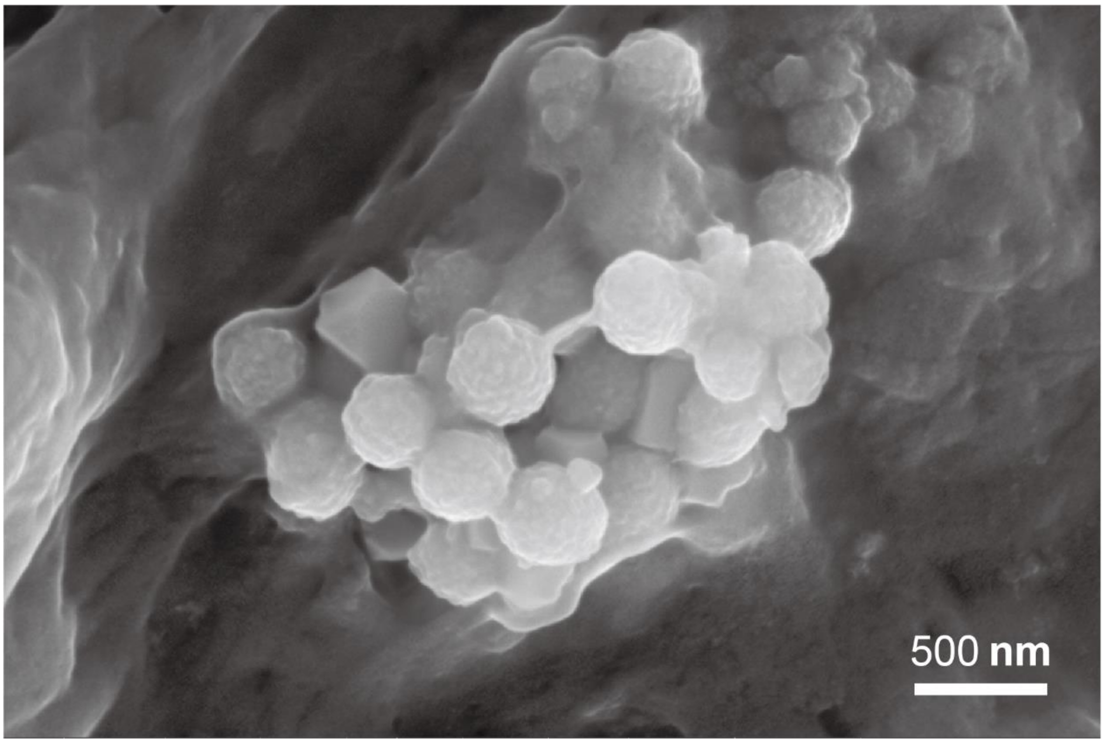

Supplementary Figure. 13. SEM image of Mn/Co_3O_4 NPs. (Scale bar: 500 nm).

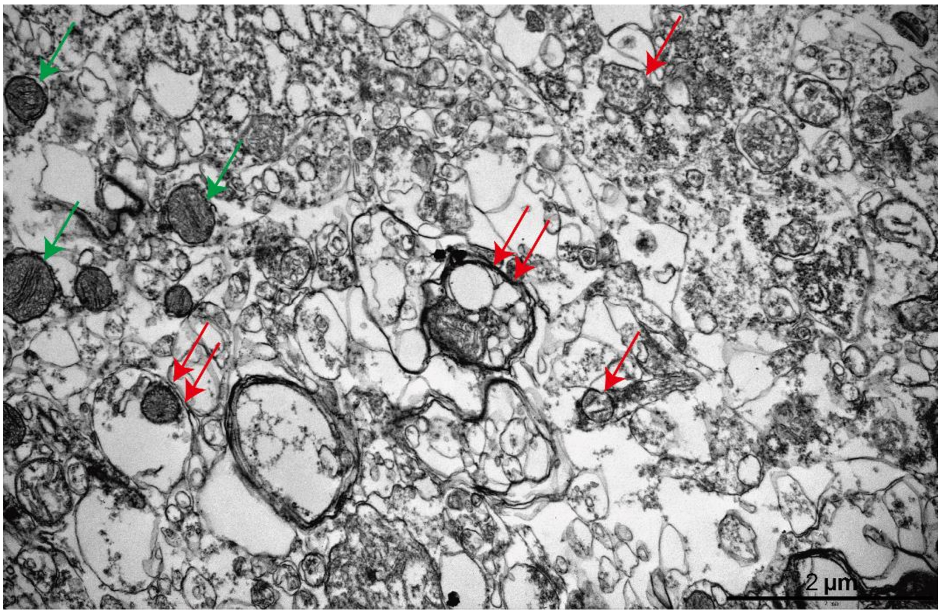

Supplementary Figure. 14. TEM image of a mouse brain section. Single arrows indicate autophagosomes, and double arrows indicate autophagic lysosomes (scale bar: 2 μm).

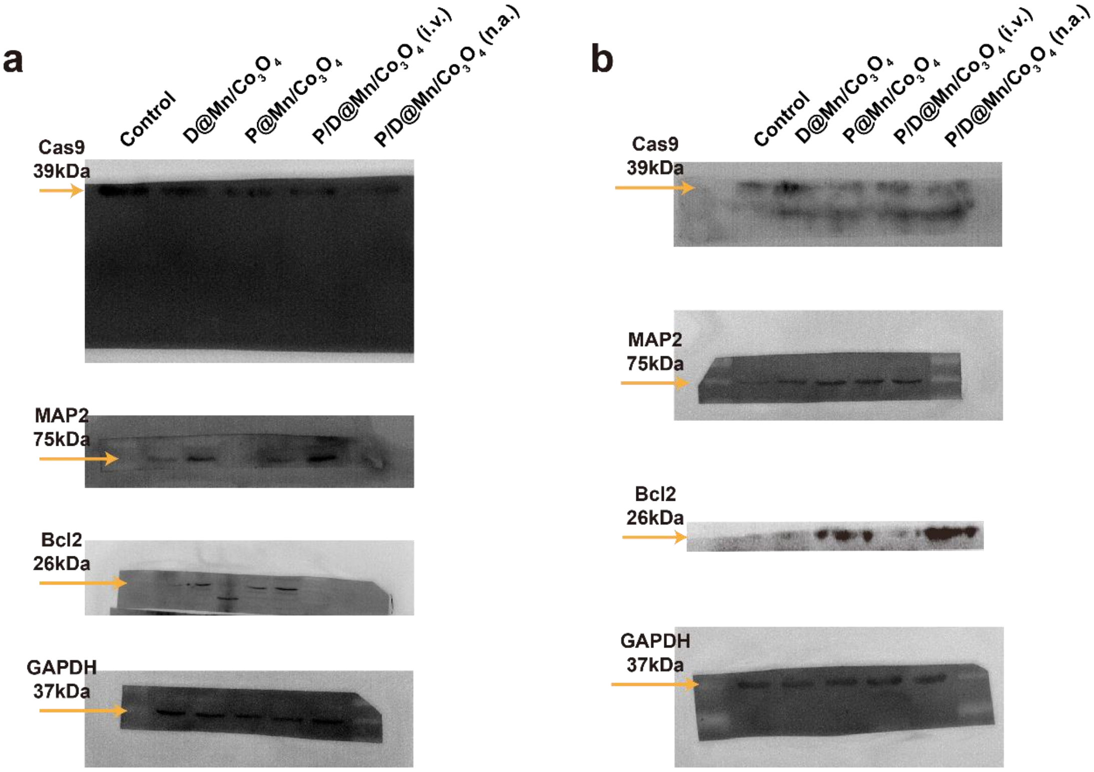

Supplementary Figure. 15. **(a)** Uncropped images of blots for Figure 4j. **(b)** Uncropped images of blots of Figure 4k.

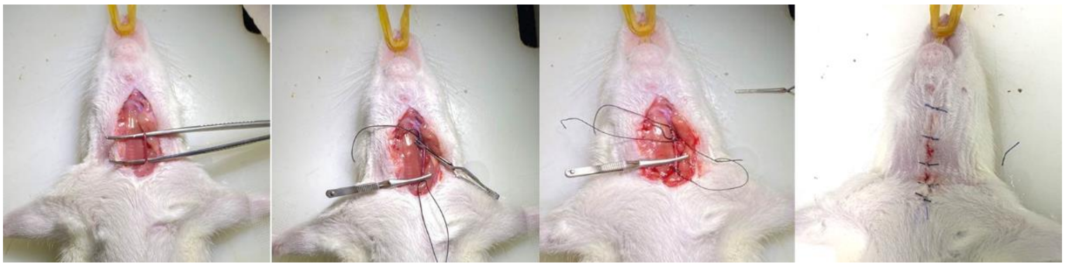

Supplementary Figure. 16. Establishment of rat stroke model by embolization method.

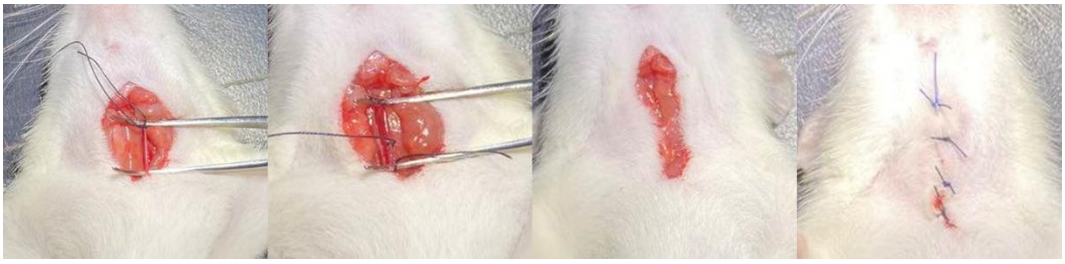

Supplementary Figure. 17. Establishment of rat VaD model by bilateral vein ligation.

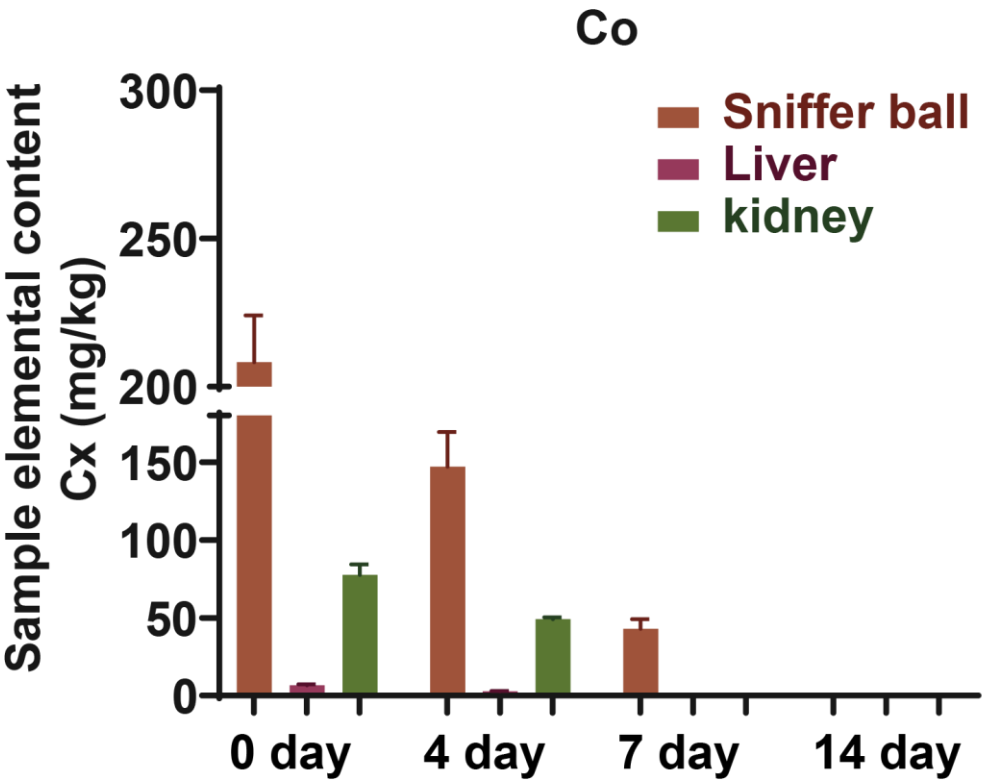

Supplementary Figure. 18. Degradation of P/D@Mn/Co_3O_4 NPs at the treatment site.
