# Supplementary material for: Engineered inhaled nanocatalytic therapy for ischemic cerebrovascular disease by inducing autophagy of abnormal mitochondria
Source: NPJ Regen Med. 2023 Aug 11;8:44. doi: 10.1038/s41536-023-00315-1 (PMC10421937; doi:10.1038/s41536-023-00315-1)
Supplement: Supplementary file 2 — nr-reporting-summary [file 41536_2023_315_MOESM2_ESM.pdf]

Corresponding author(s): Xin Zhou
Last updated by author(s): Jul 5, 2023

Reporting Summary

Nature Portfolio wishes to improve the reproducibility of the work that we publish. This form provides structure for consistency and transparency in reporting. For further information on Nature Portfolio policies, see our Editorial Policies and the Editorial Policy Checklist.

Statistics

For all statistical analyses, confirm that the following items are present in the figure legend, table legend, main text, or Methods section.

n/a	Confirmed	
☐	☒	The exact sample size (*n*) for each experimental group/condition, given as a discrete number and unit of measurement
☐	☒	A statement on whether measurements were taken from distinct samples or whether the same sample was measured repeatedly
☐	☒	The statistical test(s) used AND whether they are one- or two-sided<br>*Only common tests should be described solely by name; describe more complex techniques in the Methods section.*
☒	☐	A description of all covariates tested
☐	☒	A description of any assumptions or corrections, such as tests of normality and adjustment for multiple comparisons
☐	☒	A full description of the statistical parameters including central tendency (e.g. means) or other basic estimates (e.g. regression coefficient) AND variation (e.g. standard deviation) or associated estimates of uncertainty (e.g. confidence intervals)
☐	☒	For null hypothesis testing, the test statistic (e.g. *F*, *t*, *r*) with confidence intervals, effect sizes, degrees of freedom and *P* value noted<br>*Give P values as exact values whenever suitable.*
☒	☐	For Bayesian analysis, information on the choice of priors and Markov chain Monte Carlo settings
☒	☐	For hierarchical and complex designs, identification of the appropriate level for tests and full reporting of outcomes
☒	☐	Estimates of effect sizes (e.g. Cohen's *d*, Pearson's *r*), indicating how they were calculated

Our web collection on statistics for biologists contains articles on many of the points above.

Software and code

Policy information about availability of computer code

Data collection	*Provide a description of all commercial, open source and custom code used to collect the data in this study, specifying the version used OR state that no software was used.*
Data analysis	*Provide a description of all commercial, open source and custom code used to analyse the data in this study, specifying the version used OR state that no software was used.*

For manuscripts utilizing custom algorithms or software that are central to the research but not yet described in published literature, software must be made available to editors and reviewers. We strongly encourage code deposition in a community repository (e.g. GitHub). See the Nature Portfolio guidelines for submitting code & software for further information.

Data

Policy information about availability of data

All manuscripts must include a data availability statement. This statement should provide the following information, where applicable:

- Accession codes, unique identifiers, or web links for publicly available datasets
- A description of any restrictions on data availability
- For clinical datasets or third party data, please ensure that the statement adheres to our policy

Provide your data availability statement here.

Human research participants

Policy information about studies involving human research participants and Sex and Gender in Research.

Reporting on sex and gender	*Use the terms sex (biological attribute) and gender (shaped by social and cultural circumstances) carefully in order to avoid confusing both terms. Indicate if findings apply to only one sex or gender; describe whether sex and gender were considered in study design whether sex and/or gender was determined based on self-reporting or assigned and methods used. Provide in the source data disaggregated sex and gender data where this information has been collected, and consent has been obtained for sharing of individual-level data; provide overall numbers in this Reporting Summary. Please state if this information has not been collected. Report sex- and gender-based analyses where performed, justify reasons for lack of sex- and gender-based analysis.*
Population characteristics	*Describe the covariate-relevant population characteristics of the human research participants (e.g. age, genotypic information, past and current diagnosis and treatment categories). If you filled out the behavioural & social sciences study design questions and have nothing to add here, write "See above."*
Recruitment	*Describe how participants were recruited. Outline any potential self-selection bias or other biases that may be present and how these are likely to impact results.*
Ethics oversight	*Identify the organization(s) that approved the study protocol.*

Note that full information on the approval of the study protocol must also be provided in the manuscript.

Field-specific reporting

Please select the one below that is the best fit for your research. If you are not sure, read the appropriate sections before making your selection.

☒ Life sciences ☐ Behavioural & social sciences ☐ Ecological, evolutionary & environmental sciences

For a reference copy of the document with all sections, see nature.com/documents/nr-reporting-summary-flat.pdf

Life sciences study design

All studies must disclose on these points even when the disclosure is negative.

Sample size	n=5
Data exclusions	no data were excluded from the analyses
Replication	all attempts at replication were successful
Randomization	We used simple random assignment, i.e., all samples/organisms/participants were randomly assigned to different experimental groups with the same number of individuals in each group as possible.
Blinding	Blinding of group assignments during data collection and/or analysis

Reporting for specific materials, systems and methods

We require information from authors about some types of materials, experimental systems and methods used in many studies. Here, indicate whether each material, system or method listed is relevant to your study. If you are not sure if a list item applies to your research, read the appropriate section before selecting a response.

Materials & experimental systems

n/a	Involved in the study	
☐	☒	Antibodies
☒	☐	Eukaryotic cell lines
☒	☐	Palaeontology and archaeology
☒	☐	Animals and other organisms
☒	☐	Clinical data
☒	☐	Dual use research of concern

Methods

n/a	Involved in the study	
☒	☐	ChIP-seq
☐	☒	Flow cytometry
☒	☐	MRI-based neuroimaging

Antibodies

Antibodies used

Cas9 (S. aureus) (6H4) Mouse mAb #48989 (Cell Signaling Technology);MAP2 (D5G1) XP® Rabbit mAb #8707(Cell Signaling Technology);Bcl-2 (D17C4) Rabbit mAb (Mouse Preferred) #3498(Cell Signaling Technology);GAPDH (D16H11) XP® Rabbit mAb #5174(Cell Signaling Technology);Iba1/AIF-1 (E4O4W) XP® Rabbit mAb (Alexa Fluor® 647 Conjugate) #78060(Cell Signaling Technology);CD68 (E3O7V) Rabbit mAb (Alexa Fluor® 647 Conjugate) #17846(Cell Signaling Technology);CD206/MRC1 (E6T5J) XP® Rabbit mAb (Alexa Fluor® 594 Conjugate) #59414(Cell Signaling Technology)

Validation

Cas9 (S. aureus) (6H4) Mouse mAb #48989 (Cell Signaling Technology):Specificity/SensitivityCas9 (S. aureus) (6H4) Mouse Monoclonal Antibody detects endogenous levels of total Cas9 (Staphylococcus aureus) protein. The antibody does not cross-react with Cas9 (Streptococcus aureus), AsCpf1 (strain BV3L6) and FnCpf1 (strain U112) proteins.Species reactivity.All species expected
MAP2 (D5G1) XP® Rabbit mAb #8707(Cell Signaling Technology):MAP2 (D5G1) XP® Rabbit mAb detects endogenous levels of total MAP2 protein. Non-specific labeling of macrophages in pancreatic and mouse liver tissues is observed by immunofluorescence.Species reactivity.Mouse, RatSpecies predicted to react based on 100% sequence homology. Human
Bcl-2 (D17C4) Rabbit mAb (Mouse Preferred) #3498(Cell Signaling Technology):Specificity/SensitivityBcl-2 (D17C4) Rabbit mAb (Mouse Preferred) detects endogenous levels of total Bcl-2 protein.Species reactivity.Human, Mouse
GAPDH (D16H11) XP® Rabbit mAb #5174(Cell Signaling Technology):Specificity/SensitivityGAPDH (D16H11) XP® Rabbit mAb detects endogenous levels of total GAPDH protein.Species reactivity.Human, Mouse, Rat, MonkeySpecies predicted to be reactive based on 100% sequence homology. Pig
Iba1/AIF-1 (E4O4W) XP® Rabbit mAb (Alexa Fluor® 647 Conjugate) #78060(Cell Signaling Technology):Specificity/SensitivityIba1/AIF-1 (E4O4W) XP® Rabbit mAb (Alexa Fluor® 647 Conjugate) recognizes endogenous levels of total Iba1/AIF-1 protein.Species
CD68 (E3O7V) Rabbit mAb (Alexa Fluor® 647 Conjugate) #17846(Cell Signaling Technology):Specificity/SensitivityCD68 (E3O7V) Rabbit mAb (Alexa Fluor® 647 Conjugate) recognizes endogenous levels of total CD68 protein. Indeterminate specific staining of mouse endometrial epithelium has been observed by immunohistochemistry.Species reactivity.Mouse
CD206/MRC1 (E6T5J) XP® Rabbit mAb (Alexa Fluor® 594 Conjugate) #59414(Cell Signaling Technology):Specificity/SensitivityCD206/MRC1 (E6T5J) XP® Rabbit mAb (Alexa Fluor® 594 Conjugate) recognizes endogenous levels of total CD206/MRC1 protein. This antibody detects mouse CD206/MRC1 protein and also reacts with human CD206/MRC1, but this antibody is not recommended for immunohistochemical analysis of human tissue. Instead, the CD206/MRC1 (E2L9N) Rabbit mAb #91992 is recommended for immunohistochemical analysis of human tissue samples.Species reactivity.Human, Mouse, Rat

Flow Cytometry

Plots

Confirm that:

☒ The axis labels state the marker and fluorochrome used (e.g. CD4-FITC).

☒ The axis scales are clearly visible. Include numbers along axes only for bottom left plot of group (a 'group' is an analysis of identical markers).

☒ All plots are contour plots with outliers or pseudocolor plots.

☒ A numerical value for number of cells or percentage (with statistics) is provided.

Methodology

Sample preparation

The HT22 cells will be cultured and it is important to centrifuge each reagent before opening to ensure that the liquid is collected from the wall and mouth of the tube to avoid any loss. The 4× Binding Buffer should be diluted to 1× Binding Buffer with distilled water, and the collected cells should be gently washed with PBS and shaken. To resuspend the cells, 195 µL of 1× Binding Buffer should be added, and the cell density should be adjusted to 2-5×105 cells/ml. Then, 5 µL of Annexin V-FITC should be added to 195 µL of cell resuspension, and the mixture should be protected from light, mixed well, and incubated at room temperature for 10-15 min. Following this, the cells should be washed with 200 µL of 1X Binding Buffer, and then centrifuged at 1000 rpm for 2-5 min, with the supernatant being discarded. To perform the flow assay, the cells should be resuspended in 190 µL of 1X Binding Buffer and 10 µL of Propidium Iodide should be added. It is important to perform the flow assay within 4 h to prevent fluorescence decay.

Instrument

CytoFLEX LX

Software

FlowJo_V10

Cell population abundance

2-5×105 cells/ml

Gating strategy

1.Cell gating: Firstly, cells are identified and differentiated in the flow cytometer, selecting the cell type to be analyzed and setting a cell gate. In this experiment, HT22 cells cultured in the previous steps are used.

2.Positive control gating: A positive control gate is set to differentiate the population of cells that exhibit positivity in the Annexin V/PI double staining assay. The choice of positive control gate should be based on the positive control recommendations specific to the assay kit being used.

3.Negative control gating: To exclude false-positive cells that may appear in the experiment, a negative control gate is set. The negative control gate should be set before Annexin V/PI staining and should use a cell sample without staining.

4.Double-stained region: A double-stained region is set on the flow cytometer that covers the positive control gate and negative control gate. The experiment samples are then added to this region, and the number of stained cells is recorded.

☒ Tick this box to confirm that a figure exemplifying the gating strategy is provided in the Supplementary Information.
